# *In Vivo* Isotopic Labeling of Symbiotic Bacteria Involved in Cellulose Degradation and Nitrogen Recycling within the Gut of the Forest Cockchafer (*Melolontha hippocastani*)

**DOI:** 10.3389/fmicb.2017.01970

**Published:** 2017-10-12

**Authors:** Pol Alonso-Pernas, Stefan Bartram, Erika M. Arias-Cordero, Alexey L. Novoselov, Lorena Halty-deLeon, Yongqi Shao, Wilhelm Boland

**Affiliations:** ^1^Department of Bioorganic Chemistry, Max Planck Institute for Chemical Ecology, Jena, Germany; ^2^Department of Evolutionary Neuroethology, Max Planck Institute for Chemical Ecology, Jena, Germany; ^3^Institute of Sericulture and Apiculture, College of Animal Sciences, Zhejiang University, Hangzhou, China

**Keywords:** *Melolontha hippocastani*, nitrogen recycling, cellulose degradation, gut bacteria, symbiotic bacteria, Illumina-SIP, IRMS

## Abstract

The guts of insects harbor symbiotic bacterial communities. However, due to their complexity, it is challenging to relate a specific symbiotic phylotype to its corresponding function. In the present study, we focused on the forest cockchafer (*Melolontha hippocastani*), a phytophagous insect with a dual life cycle, consisting of a root-feeding larval stage and a leaf-feeding adult stage. By combining *in vivo* stable isotope probing (SIP) with ^13^C cellulose and ^15^N urea as trophic links, with Illumina MiSeq (Illumina-SIP), we unraveled bacterial networks processing recalcitrant dietary components and recycling nitrogenous waste. The bacterial communities behind these processes change between larval and adult stages. In ^13^C cellulose-fed insects, the bacterial families Lachnospiraceae and Enterobacteriaceae were isotopically labeled in larvae and adults, respectively. In ^15^N urea-fed insects, the genera *Burkholderia* and *Parabacteroides* were isotopically labeled in larvae and adults, respectively. Additionally, the PICRUSt-predicted metagenome suggested a possible ability to degrade hemicellulose and to produce amino acids of, respectively, ^13^C cellulose- and ^15^N urea labeled bacteria. The incorporation of ^15^N from ingested urea back into the insect body was confirmed, in larvae and adults, by isotope ratio mass spectrometry (IRMS). Besides highlighting key bacterial symbionts of the gut of *M. hippocastani*, this study provides example on how Illumina-SIP with multiple trophic links can be used to target microorganisms embracing different roles within an environment.

## Introduction

Symbiotic associations between insects and gut-dwelling microorganisms, especially bacteria, have long been known (Baumberger, 1919), but it was not until the development of next-generation sequencing techniques that we realized the true extent of the diversity and complexity of such microbial communities (Shi et al., 2010). Only one major phylotype is present in insects of the Alydae family, whereas termites can harbor hundreds of major bacterial taxa (Engel and Moran, 2013). A relationship between community composition and host insect taxon and diet has been demonstrated (Colman et al., 2012; Jones et al., 2013; Yun et al., 2014; Guerrero et al., 2016), suggesting crucial roles of the symbionts with regard to insect physiology (Potrikus and Breznak, 1981; Douglas, 2009; Watanabe and Tokuda, 2010).

Cleveland demonstrated for the first time that termite gut symbionts are essential for the digestion of cellulose and for the viability of the host (Cleveland, 1924). Since then, the contribution of insect symbionts in cellulose digestion (Rössler, 1961; Bayon and Mathelin, 1980; Martin, 1983; Anand et al., 2010) and, more recently, in the recycling of nitrogen (Potrikus and Breznak, 1981; Sasaki et al., 1996; Sabree et al., 2009; Thong-On et al., 2012; Ayayee et al., 2014) have been object of extensive research. In insects possessing specialized hindguts chambers such as termites or scarabaeid beetles, cellulose breakdown occurs thanks to the combined action of host- and microbe-secreted enzymes (Watanabe and Tokuda, 2010; Calderon-Cortes et al., 2012). To which extent the host contributes to the recycling of nitrogen is still unclear; however, a shared pathway has been reported in the cockroach *Blatella germanica* (Patiño-Navarrete et al., 2014).

Nonetheless, the insect digestive tract is a complex ecosystem that renders challenging to relate specific microorganisms to their role within the community. Studies involving bacterial isolation allowed the identification of some cellulolytic and uricolytic bacteria (Bridges, 1981; Anand et al., 2010; Huang et al., 2012; Morales-jiménez et al., 2013), but the lack of culturability of the majority of gut microorganisms and the impossibility of accurately reproducing in artificial media the natural conditions of the digestive tract make cumbersome to assess the real significance of such *in vitro* observations in an *in vivo* scenario. The combination of stable isotope probing (SIP) with culture-independent methods of microorganism identification has proven to be a reliable and straightforward approach that allows to link a particular function to specific community members (Lu and Conrad, 2005; Bell et al., 2011; Reichardt et al., 2011; Wüst et al., 2011). By introducing the isotopically labeled substrate of interest into the otherwise unmodified environment, it is possible to disclose an organism's ability to incorporate the heavy isotope into its nucleic acids, thus directly relating certain bacterial taxa to the use of a specific compound (Dumont and Murrell, 2005). In order to increase sensitivity, SIP can be coupled with high throughput amplicon sequencing methods such as Pyrosequencing or Illumina (Aoyagi et al., 2015). This combination has been applied to determine the relevant microorganisms in diverse environments (Paes et al., 2015; Liu et al., 2017) and within the gut of mammals (Berry et al., 2013; Godwin et al., 2014) or insects (Shao et al., 2014).

Our object of study is the gut symbiotic bacteria of the forest cockchafer (*Melolonthahippocastani*), a beetle belonging to the Scarabaeidae family. During its entire life cycle, this insect feeds on living plants, either on the roots during the underground larval stage or on the leaves in the aboveground adult stage, causing significant damage to vegetation during occasional population outbreaks (Jackson and Klein, 2006). The composition of the intricate bacterial community that populates the digestive tract of *M. hippocastani* remained unknown until the works of Arias-Cordero et al. (2012) and Alonso-Pernas et al. (2017), as previous studies in scarabaeid microbiome focused on other beetles (Egert et al., 2003; Zhang and Jackson, 2008; Franzini et al., 2016) or its closely related species, *Melolontha melolontha* (Egert et al., 2005). These works have shown that, at the class level, the taxonomic composition of the gut community of *M. hippocastani* remains constant across the entire insect life cycle, notwithstanding the variation in habitat and nourishment between larval and adult stages (Arias-Cordero et al., 2012; Alonso-Pernas et al., 2017).

Since these purely descriptive studies did not allow inference about the function of the gut symbionts, in the present study we combined DNA isotopic labeling with Illumina MiSeq in order to shed some light on the actual role of the gut bacteria in key nutritional processes of phytophagous insects, namely the degradation of cellulose and the recycling of nitrogen. For those purposes, we fed the insects with diet mixed with either ^13^C cellulose, or ^15^N urea, a component of insect nitrogenous waste (Bursell, 1967; Ayayee et al., 2014). Considering the constancy of *M. hippocastani* symbiotic community, we expected to unmask a taxonomic shift in the active microbial players coupled with the transition from larval to adult stage, as a result of adaptation to the radically new diet. By isotope ratio mass spectrometry (IRMS) analyses of host tissues, we aimed to detect the incorporation of ^15^N from urea into the insect body, thus confirming the existence of a nitrogen-recycling mechanism (Potrikus and Breznak, 1981). Additionally, by introducing the obtained sequencing data of the 16S rRNA genes to the PICRUSt software, which predicts the functional composition of a metagenome using marker gene data and reference genomes (Langille et al., 2013), we produced predictions of the mechanisms that might be behind the above-mentioned processes.

## Materials and methods

### Insect collection, feeding, and DNA extraction

Second-instar (L2) larvae were collected in a deciduous forest next to Pfungstadt (Germany, 49°49′44″ N 8°36′17″ E) in June 2016. Adult insects were collected in a deciduous forest next to Hanau (Germany, 50°07′02″ N 8°59′26″ E) in May 2016. Insects were transported to the laboratory in boxes with soil or three leaves. ^12^C cellulose, ^14^N urea, and ^15^N urea were purchased from Sigma-Aldrich (St. Louis, MO, USA). ^13^C cellulose purified from potato was purchased from IsoLife (Wageningen, The Netherlands). Upon arrival, larvae were transferred to autoclaved sterile plant soil and fed with grated carrot mixed with the experimental compound at a final concentration in the diet of 4 mM (urea) (Ayayee et al., 2014) and 60 mM (cellulose). Optimal cellulose concentration in the diet was estimated based on data from previous labeling experiments with ^13^C glucose (Alonso-Pernas, unpublished data). Adults were fed with ethanol-sterilized oak leaves overlaid with an aqueous solution of urea or a cellulose suspension at the same final concentrations mentioned above. For each substrate and life stage, a treatment group was fed with isotopically labeled compound, and a control group was fed with unlabeled compound. A group was composed of three insects. Each insect was fed separately in an individual compartment within a climate chamber simulating natural conditions of light, temperature and humidity, for 7 days (urea) and 5 days (cellulose). By using long feeding time we aimed to unveil not only the primary degraders of the substrate, but also the subsequent secondary utilizers that process the derived metabolites (Neufeld et al., 2007; Shao et al., 2014). After feeding, insects were frozen and stored at −80°C until further processing. Before dissection, insects were thawed on ice and surface-sterilized by being soaked briefly in 70% ethanol and sterile distilled water. Dissection was carried out in phosphate-buffered saline (PBS) solution, on ice. Composition of PBS solution used (per liter) is as follows: 8 g NaCl, 0.2 g KCl, 1.44 g Na_2_HPO_4_, 0.24 g KH_2_PO_4_ (pH 7.4). Whole guts were carefully extracted and dried in a Concentrator 5301 Speedvac (Eppendorf, Hamburg, Germany) at 45°C for 90 min, and then crushed with a sterile pestle. 5 mg of dry powdered gut tissue was used for DNA extraction with the MasterPure™ Complete DNA and RNA Purification Kit (Illumina, San Diego, CA, USA) according to manufacturer's instructions. The concentration and purity of extracted DNA were checked with NanoDrop One spectrophotometer (Thermo Fisher Scientific, Waltham, MA, USA). DNA was stored at −20°C until further processing.

### DNA separation in CsCl density gradient and fractionation

CsCl gradients were prepared as described (Neufeld et al., 2007). In short, ~2 μg of purified DNA was mixed with 4.8 ml of 7.163 M CsCl aqueous solution and the corresponding volume of gradient buffer (0.1 M Tris, 0.1 M KCl, and 1 mM EDTA) to achieve a final density of 1.725 g/ml. Solution was transferred to 5.1 ml polyallomer tubes (Beckham Coulter, Brea, CA, USA) and centrifuged in an Optima™ L-90K ultracentrifuge fitted with a NVT 90 rotor (Beckham Coulter, Brea, CA, USA) for 40 h at 173,000 g for cellulose gradients, and 66 h at 150,000 g for urea gradients, as longer centrifugation times at lower speeds enhance DNA separation in 15N-labeled gradients (Cadisch et al., 2005). All samples from the same substrate were set up in the same CsCl batch and run in parallel to minimize potential variations. After centrifugation, 400 μL (cellulose) and 200 μL (urea) fractions were collected drop-wise from the bottom of the tube (total of 12 fractions for cellulose gradients, 24 for urea gradients). The density of each fraction was determined by weighing per triplicate a volume of 100 μL in a fine-scale balance. DNA was precipitated with polyethylene glycol (PEG) 6000 (VWR International, Radnor, PA, USA), washed twice with 70% ethanol and resuspended in sterile double-distilled water.

### DNA concentration measurement and qPCR of selected density ranges

**Figure 1:**
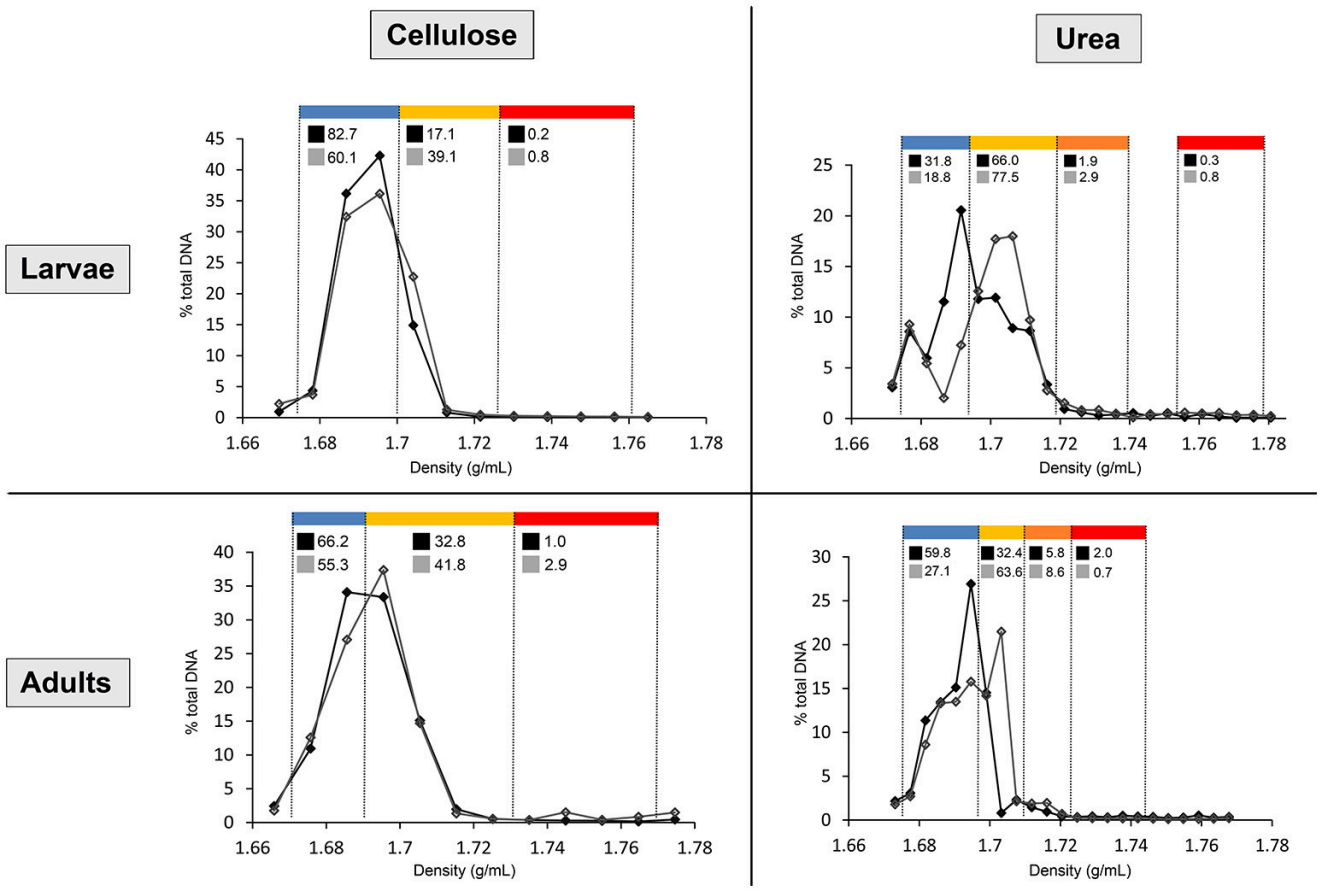
Distribution of the DNA in cellulose and urea gradients, in larvae and adults. Line chart shows the percentages of total gradient DNA contained in each fraction of control gradient (black line, full markers) and isotopically labeled gradient (gray line, empty markers). Density ranges constituting each pooled fraction are indicated in blue (upper fraction), yellow (medium fraction in cellulose gradients, upper medium fraction in urea gradients), orange (lower medium fraction in urea gradients), and red (lower fraction). Numbers above the line chart show the percentages of total gradient 16S rRNA gene copy number contained in each pooled fraction of control gradient (black square) and labeled gradient (gray square).

The DNA-density profile for each gradient was assessed using the Helixyte Green™ Nucleic Acid Stain (Bioquest, Bengaluru, India), according to the protocol provided by the manufacturer. The fluorescence was measured with an Infinite F200 PRO plate reader (Tecan, Männedorf, Switzerland), and DNA concentration for each fraction was inferred based on a standard curve. For each experimental compound, equal density ranges showing visible difference in amount of DNA between treatments were selected, and fractions within each range were grouped together in pooled fractions (upper, medium (upper medium and lower medium in urea gradients) and lower) (Figure 1). The 16S rRNA gene copy number for each pooled fraction was determined by quantitative real-time PCR (qPCR) (Lueders et al., 2004) using the 16S rRNA gene specific primers Bact 519F (5′-CAG CMG CCG CGG TAA NWC-3′) and Bact 907R (5′-CCG TCA ATT CMT TTR AGT T-3′) (Stubner, 2002). Reactions contained 2 μL of template DNA, primers at a concentration of 0.3 mM each and 1x Brilliant III SYBR® Green QPCR Master Mix (Agilent, Santa Clara, CA, USA), making a total volume of 20 μL. The program consisted of a 95°C hold for 5 min followed by 40 cycles of 45 s at 95°C, 30 s at 50°C and 50 s at 72°C. PCRs were carried out in a CFX96™ Real-Time PCR Detection System (BioRad, Hercules, CA, USA). *E. coli* genomic DNA was used to relate quantitative cycle values to the 16S rRNA gene copy number of each reaction (Supplementary Figure 1). In order to correct for differences in amount of recovered DNA and allow comparison between control and labeled gradients, fraction DNA and 16S rRNA gene copy number were normalized as percentage of, respectively, the total amount of DNA or 16S rRNA copy number of all the fractions within a gradient.

### Illumina sequencing, data analysis, and metagenome reconstruction

Pooled fractions were submitted for 16S rRNA gene Illumina MiSeq to Molecular Research Laboratory (www.mrdnalab.com; Shallowater, TX, USA). In short, the 16S rRNA gene V4 variable region was amplified using the PCR primers 28F (5′-GAG TTT GAT CNT GGC TCA G-3′) and 519R (5′-GTN TTA CNG CGG CKG CTG-3′). After amplification, PCR products were checked in 2% agarose gel and purified using Agencourt AMPure beads (Agencourt Bioscience Corporation, Beverly, MA, USA). The purified PCR products were used to prepare the DNA library following the Illumina MiSeq DNA library preparation protocol using the MiSeq reagent kit V3 (2X300 bp) for paired-end reads following the manufacturer's guidelines. The quality control and analysis of Illumina reads was done in QIIME version 1.8.0 (Caporaso et al., 2010a). Low-quality reads (quality cut-off = 25) and sequences <200 bp in length were removed, and the remaining reads were denoised using the “denoiser” algorithm as implemented in QIIME (Reeder and Knight, 2010). Denoised high-quality reads were clustered into operational taxonomic units (OTUs) using the *pick_open_reference_otus.py* script and uclust (Edgar, 2010) with 97% nucleotide identity cut-offs. For each OTU, the most abundant sequence was chosen as a representative sequence and aligned to the newest Greengenes core set available (http://greengenes.lbl.gov/) (McDonald et al., 2012) using PyNast (Caporaso et al., 2010b). RDP classifier was used for taxonomy assignment (Wang et al., 2007). An OTU table was constructed describing the abundance of each bacterial phylotype in each pooled fraction. Abundances were normalized as percentages of total sequences in order to correct for differences in library sizes. Based on previous research, activity of a bacterial taxon was defined as its increase in percentage of relative abundance (enrichment) in a dense pooled fraction (medium or lower) of the labeled gradient compared to the same pooled fraction in the control (unlabeled) gradient (Lu and Conrad, 2005; Wüst et al., 2011; Shao et al., 2014):

$$\begin{array}{l} A=\left[\frac{tNS_{labeled\;pooled\;fraction}}{NS_{labeled\;pooled\;fraction}}\;\cdot \;100\right]\;-\;\left[\frac{tNS_{control\;pooled\;fraction}}{NS_{control\;pooled\;fraction}}\;\cdot \;100\right] \end{array}$$

A stands for activity, tNS stands for number of sequences belonging to a certain taxon, NS stands for total number of sequences. One activity value was obtained for each dense pooled fraction (two for cellulose gradients, three for urea gradients). Identification of taxa that were significantly enriched in dense, labeled pooled fractions (active taxa) was carried out using a Fisher Exact Test with FDR correction in METASTATS (White et al., 2009). The significance threshold was set at *p* = 0.05, but taxa active with *p* < 0.1 were also considered for discussion as tending to be significant. Heatmaps representing level of activity were constructed using the MultiExperiment Viewer (MeV) software (Saeed et al., 2003). Phylogenetic trees were calculated using the maximum likelihood method (General Time Reversible model) with 1,000 bootstrap replicates in MEGA6 (Tamura et al., 2013). The bacterial metagenome of medium and lower pooled fractions was reconstructed using the PICRUSt software (Langille et al., 2013), using as the input file a PICRUSt-compatible closed-reference OTU table generated from the above-mentioned open-reference OTU table with the *filter_otus_from_otu_table.py* script. Non-labeled families were filtered out of the OTU table using the *filter_taxa_from_otu_table.py* script. 16S rRNA copy numbers per OTU were normalized with the *normalize_by_copy_number.py* script and IMG database information. The metagenome inference was performed with the *predict_metagenomes.py* script, using the normalized OTU table as input. NSTI values, a measure of prediction uncertainty, were also calculated (Supplementary Table 1). Certain KEGG Orthologs were selected according to their relationship with the experimental substrate (lignocellulose digestion for cellulose, nitrogenous waste degradation and amino acid production for urea). Calculations of the enrichment of KEGG Orthologs in dense isotopically-labeled pooled fractions, reflecting their presence in labeled bacterial families, and construction of heatmaps were done as described above for activity of bacterial taxa.

### Determination of the level of ^15^N isotopic enrichment of insect tissues

To determine the level of ^15^N enrichment in the insect tissue, larvae, and adults were dissected as described above to excise the tissues of interest (gut, fat bodies, and muscular tissue). Tissues were rinsed in sterile PBS before being dried and crushed. For each life stage, three control insects fed with ^14^N urea and three treated insects fed with ^15^N urea were used for the measurement. Three technical replicates of each biological replicate were analyzed. The abundance of the ^15^N was determined by a coupled elemental analyzer/isotope ratio mass spectrometry (EA/IRMS) and is given in δ-notation (*vide infra*). About 0.3 mg of dry, powdered tissue was weighed with an ultra-micro balance (UMX2, Mettler-Toledo) in small 0.04 ml tin capsules (3.5 × 5 mm, HEKATech, HE 24005300). The capsules were sealed and combusted (oxidation at 1,020°C, reduction at 650°C) in a constant helium stream (80 ml/min) quantitatively to CO_2_, N_2_, and H_2_O using an elemental analyzer (EuroEA CN2 dual, HEKAtech GmbH, Wegberg, Germany). After passing through a water trap (MgClO_4_) the gases were separated chromatographically at 85°C and transferred via an open split to a coupled isotope ratio mass spectrometer (IsoPrime, Micromass, Manchester, UK). Isotope ratios were generally calculated as:

$$\begin{array}{l} \delta^{N}E=\left[\frac{\left(R_{sample}-R_{standard}\right)}{R_{standard}}\right]-1 \end{array}$$

δ values usually are small numbers. Hence, they are commonly multiplied by 1,000 and communicated in ‰units or mUr (Brand and Coplen, 2012). N is the heavy isotope of the element E, R is the ratio of heavy to light isotope (^15^N/^14^N) of the sample and the standard, respectively. δNE is the relative deviation of the heavy to light isotope ratio from the international standard (air-N_2_ for nitrogen). Samples were measured against our laboratory working standard alice-1 (acetanilide, δ^15^N = –1.44 ± 0.12‰), which has been calibrated for δ15N by a two-point normalization using IAEA-N1 (+0.43‰), and IAEA-N2 (+20.40‰) (Böhlke and Coplen, 1995). Empty tin capsules were used as blanks. A caffeine standard (cafice-1, δ^15^N = −4.01 ± 0.10‰) was analyzed together with the samples as quality assurance reference material for long-term performance monitoring of the whole analytical setup (Werner and Brand, 2001). δ^15^N-values of enriched samples are not corrected for m/z = 30 trace (^15^N_2_). Since number of biological replicates (*n* = 3) was too small to test for normal distribution, the statistical significance of differences in δ values was calculated using the Mann–Whitney U test (significance threshold *p* = 0.05) in Prism 4 software (GraphPad Software Inc., La Jolla, CA, USA). Data are given as mean +/− SEM (Standard Error of Mean).

### Nucleotide sequence accession numbers

The Illumina raw sequencing data were deposited at the NCBI GenBank Short Read Archive under accession numbers SRR5296204 (^12^C and ^13^C cellulose gradients) and SRR5296203 (^14^N and ^15^N urea gradients).

## Results

### Isopycnic centrifugation and separation of light and heavy DNA

After centrifugation, between 500 and 1,000 ng of DNA were recovered from the gradients by PEG precipitation. In order to normalize the variation in amount of recovered DNA between gradients, fraction's DNA concentration and 16S rRNA gene copy number are expressed as percentages of total amount within the gradient. For urea, shifting of the DNA toward dense parts (>1.69 g/mL) of the labeled gradient (^15^N) compared to control (^14^N) is already observable in the quantification carried out with Helyxyte green. qPCR performed with bacteria-specific primers clarified the increase of 16S rRNA gene copy number in dense pooled fractions (medium and lower) of the isotopically labeled gradient (^13^C or ^15^N) in comparison to the unlabeled control gradient (^12^C or ^14^N), confirming successful labeling for both substrates (Figure 1).

### Illumina MiSeq analysis of bacterial diversity in pooled fractions and assessment of relative activity

After processing raw Illumina MiSeq data, 145,737 (cellulose larvae) 134,580 (cellulose adults) 367,589 (urea larvae) 531,465 (urea adults) high quality reads were obtained. The sequence analysis showed an increase of the relative abundance of particular bacterial taxa in the dense pooled fractions (medium and lower) of the isotopically labeled gradient (^13^C or ^15^N) compared to those in the control gradient (^12^C or ^14^N). This indicates the incorporation of the heavy isotope into the bacterial DNA (labeling). Based on the variation in relative abundance between dense pooled fractions of the labeled gradient and those of the control gradient, the activity value of each bacterial taxon for each experimental substrate was determined.

**Figure 2:**
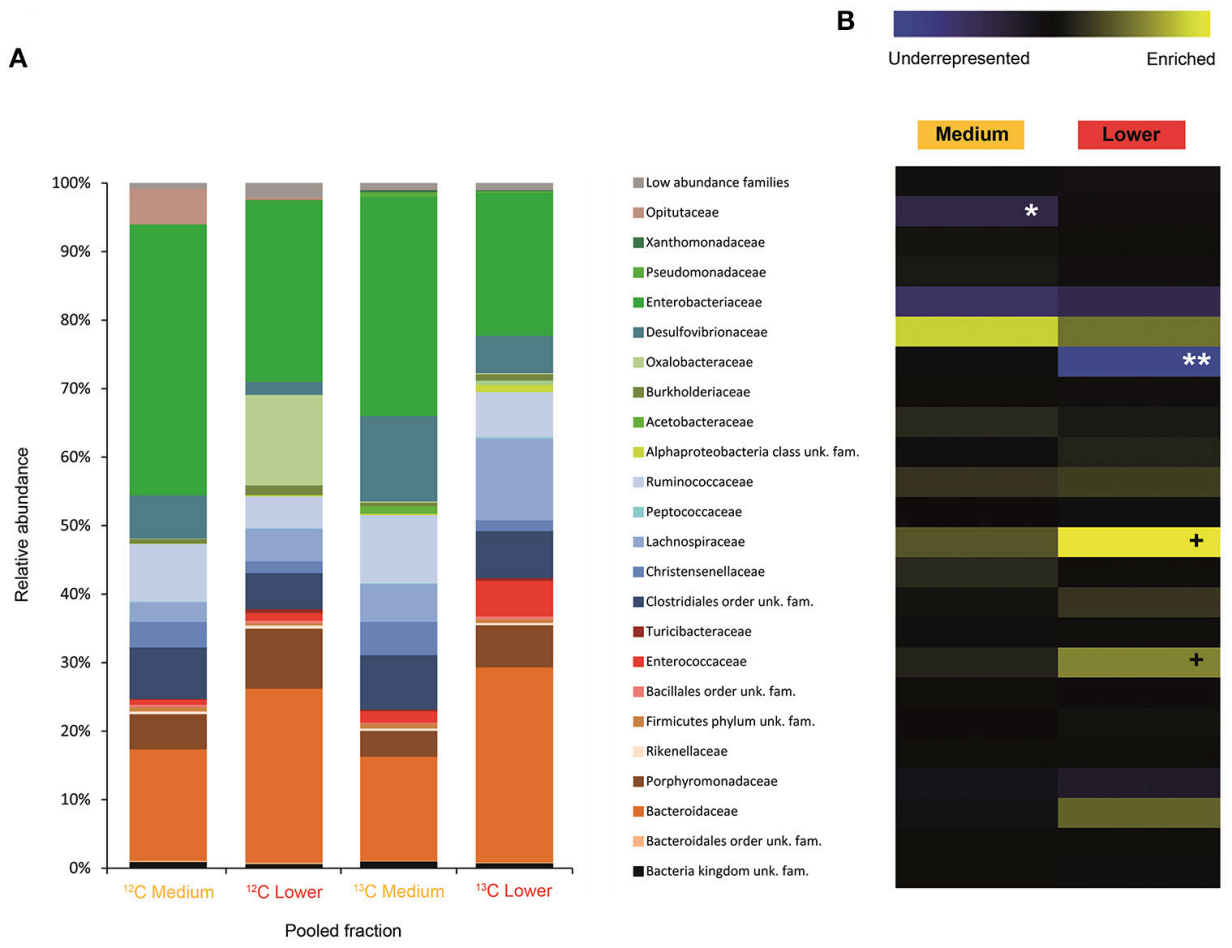
Result of the Illumina MiSeq sequencing of the gradient constructed with DNA from larvae fed on diet supplemented with cellulose (145, 737 sequences in total). The relative abundance of bacterial families in dense pooled fractions of ^12^C and ^13^C gradients, in percentage of total sequences, is displayed in **(A)**. The enrichment or underrepresentation of bacterial families in the medium and lower pooled fractions of the ^13^C gradient compared to the same fractions in the ^12^C gradient, as a measure of the activity in the processing of cellulose, is shown in **(B)**. Blue represents underrepresentation (inactive families), while yellow represents enrichment (active families). ^**^p < 0.01; ^*^p < 0.05; + *p* < 0.1.

**Figure 3:**
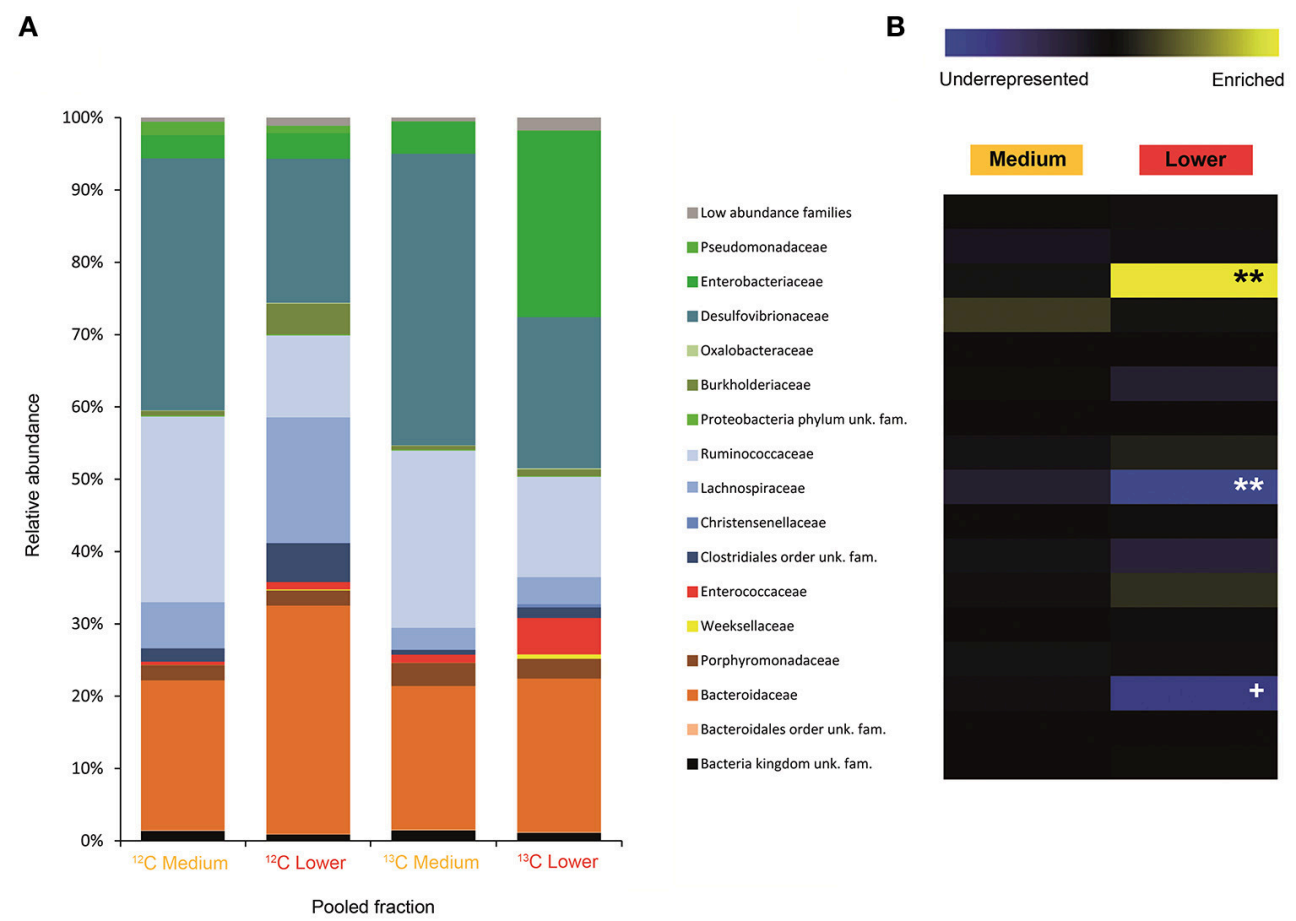
Result of the Illumina MiSeq sequencing of the gradient constructed with DNA from adults fed on diet supplemented with cellulose (134, 580 sequences in total). The relative abundance of bacterial families in dense pooled fractions of ^12^C and ^13^C gradients, in percentage of total sequences, is displayed in **(A)**. The enrichment or underrepresentation of bacterial families in the medium and lower pooled fractions of the ^13^C gradient compared to the same fractions in the ^12^C gradient, as a measure of the activity in the processing of cellulose, is shown in **(B)**. Blue represents underrepresentation (inactive families), while yellow represents enrichment (active families). ^**^p < 0.01; ^+^ *p* < 0.1.

In larvae fed on diet supplemented with cellulose, the detected families showing activity were Lachnospiraceae and Enterococcaceae (*p* < 0.1), Desulfovibrionaceae, Ruminococcaceae, Bacteroidaceae, unclassified families in the order Clostridiales, unclassified families in the class Alphaproteobacteria, Christensenellaceae, and Acetobacteraceae (non-significant) (Figure 2). However, none of them achieved statistically significant labeling (*p* < 0.05), possibly due to occurence of a strong interspecific competition for the experimental substrate, slow growth and/or usage of other carbon sources besides cellulose. In adults, detected active families were Enterobacteriaceae (*p* < 0.01), and Enterococcaceae, Desulfovibrionaceae, Ruminococcaceae, and Porphyromonadaceae (non-significant) (Figure 3). The highly significant activity of the Enterobacteriaceae family suggests that, in contrast to larvae, a single family might undertake the degradation of cellulose in the adult gut. Interestingly, no appreciable labeling of the Enterobacteriaceae family was observed in larvae. Lachnospiraceae, the family showing the highest activity value in larvae, is significantly underrepresented in the lower pooled fraction of labeled adult gradient (*p* < 0.01). This suggests the occurrence of an expected taxonomic shift in the active bacteria across the host's developmental stages. Not all the OTUs could be classified at the genus level. Nevertheless, among the identified genera, the genus *Trabulsiella* (Enterobacteriaceae family) showed the highest activity in larvae (*p* < 0.05). In adults, the genus *Enterococcus* (Enterococcaceae family) had the highest activity, although not statistically significant (Table 1). Maximum likelihood trees of the most active of the identified genera with closely related NCBI retrieved sequences are shown in Supplementary Figure 2.

**Table 1:** Bacterial genera showing the highest activity values among the identified genera in larvae and adults fed on diet supplemented with cellulose.

**Cellulose**							
**Larvae**				**Adults**			
**Family**	**Genus**	**Med**	**Low**	**Family**	**Genus**	**Med**	**Low**
Enterobacteriaceae	*Trabulsiella*	7.0^*^	4.0	Enterococcaceae	*Enterococcus*	−0.1	2.8
Porphyromonadaceae	*Parabacteroides*	1.9	3.1	Porphyromonadaceae	*Parabacteroides*	1.0	0.7
Bacteroidaceae	*Bacteroides*	−1.0	3.0	Enterobacteriaceae	*Serratia*	0.0	1.4
Acetobacteraceae	*Gluconobacter*	1.1	0.6	Enterobacteriaceae	*Erwinia*	0.0	1.2
Enterobacteriaceae	*Serratia*	0.8	0.4				

*Activity values of each genus in medium and lower pooled fractions is showed in Med and Low columns, respectively*.

* *p < 0.05*.

**Figure 4:**
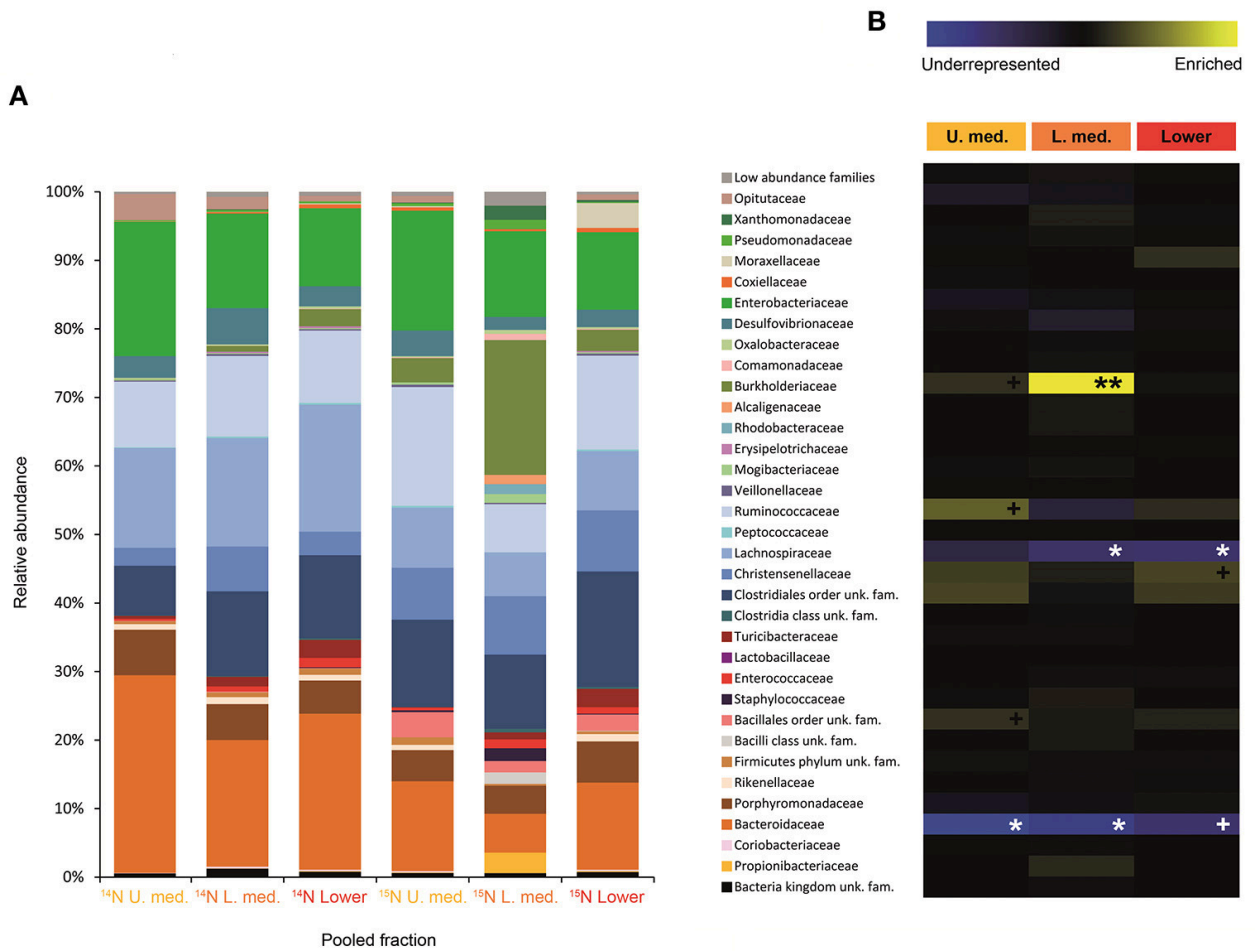
Result of the Illumina MiSeq sequencing of the gradient constructed with DNA from larvae fed on diet supplemented with urea (367, 589 sequences in total). The relative abundance of bacterial families in dense pooled fractions of ^14^N and ^15^N gradients, in percentage of total sequences, is displayed in **(A)**. The enrichment or underrepresentation of bacterial families in the medium and lower pooled fractions of the ^15^N gradient compared to the same fractions in the ^14^N gradient, as a measure of the activity in the processing of urea, is shown in **(B)**. Blue represents underrepresentation (inactive families), while yellow represents enrichment (active families). ^**^p < 0.01; ^*^p < 0.05; ^+^*p* < 0.1.

**Figure 5:**
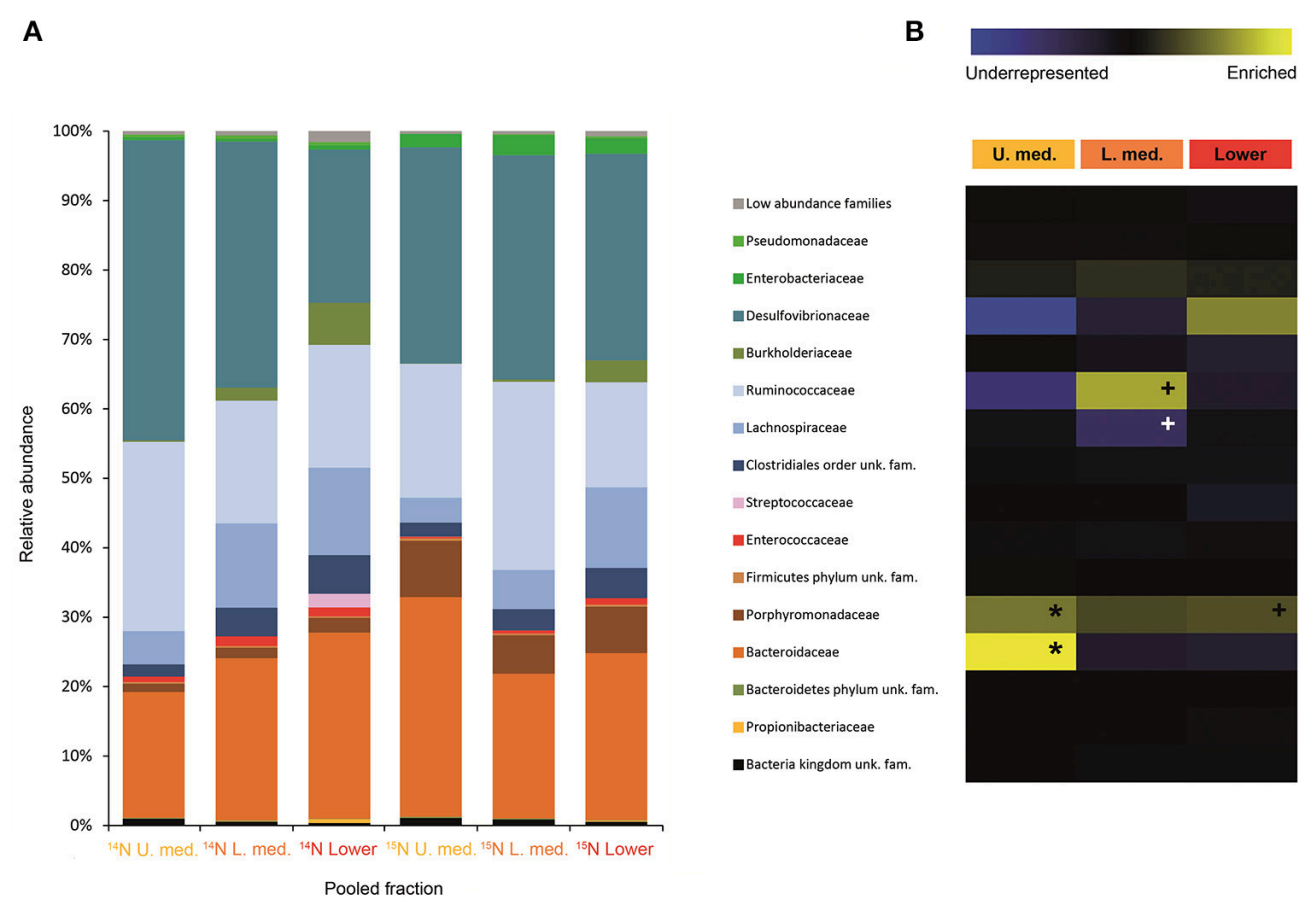
Result of the Illumina MiSeq sequencing of the gradient constructed with DNA from adults fed on diet supplemented with urea (531, 465 sequences in total). The relative abundance of bacterial families in dense pooled fractions of ^14^N and ^15^N gradients, in percentage of total sequences, is displayed in **(A)**. The enrichment or underrepresentation of bacterial families in the medium lower pooled fractions of the ^15^N gradient compared to the same fractions in the ^14^N gradient, as a measure of the activity in the processing of urea, is shown in **(B)**. Blue represents underrepresentation (inactive families), while yellow represents enrichment (active families). ^*^p < 0.05; ^+^ *p* < 0.1.

Labeling of certain bacterial families was also accomplished using urea as a trophic link, indicating the ability of gut bacterial symbionts to incorporate nitrogen from the insect's waste compounds into nucleic acids. A high number of bacterial families in the larvae were enriched in the medium and lower pooled fractions of the ^15^N gradient; following are the ones with higher activities values: Burkholderiaceae (*p* < 0.01), Christensenellaceae, Ruminococcaceae and unclassified families in the order Bacillales (*p* < 0.1), unclassified families in the order Clostridiales, Moraxellaceae, Propionibacteriaceae, Xanthomonadaceae, Staphylococcaceae, and unclassified families in the class Bacilli (non-significant) (Figure 4). The detected active bacterial families in the adults were Porphyromonadaceae and Bacteroidaceae (*p* < 0.05), Ruminococcaceae (*p* < 0.1), Desulfovibrionaceae, and Enterobacteriaceae (non-significant) (Figure 5). Again, bacteria were differently labeled when comparing larvae and adults, suggesting that the taxonomic shift in active communities also happens in symbionts involved in urea processing. Among the OTUs that could be classified to the genus level, *Burkholderia* sp. (99% of the Burkholderiaceae family sequences) showed highly significant activity in larvae (*p* < 0.01). *Parabacteroides* sp. (99% of the Porphyromonadaceae family sequences) and *Bacteroides* sp. (100% of the Bacteroidaceae family sequences) also achieved statistically significant activities in adults (*p* < 0.05) (Table 2). Maximum likelihood trees of the most active of the identified genera with closely related NCBI retrieved sequences are shown in Supplementary Figure 2.

**Table 2:** Bacterial genera showing the highest activity values among the identified genera in larvae and adults fed on diet supplemented with urea.

**Urea**									
**Larvae**				**Adults**					
**Family**	**Genus**	**Umed**	**Lmed**	**Low**	**Family**	**Genus**	**Umed**	**Lmed**	**Low**
Burkholderiaceae	*Burkholderia*	3.4	18.4^**^	1.0	Porphyromonadaceae	*Parabacteroides*	6.4^*^	3.9	4.4 +
Moraxellaceae	*Enhydrobacter*	0.2	0.0	3.6	Bacteroidaceae	*Bacteroides*	13.5^*^	−2.3	−2.7
Enterobacteriaceae	*Erwinia*	0.9	1.8	0.5	Enterobacteriaceae	*Gluconacetobacter*	1.0	1.3	1.0
Propionibacteriaceae	*Propionibacterium*	0.1	2.9	0.0					
Porphyromonadaceae	*Dysgonomonas*	1.1	0.4	1.4					
Xanthomonadaceae	*Stenotrophomonas*	0.1	2.0	0.3					
Staphylococcaceae	*Staphylococcus*	0.3	1.8	0.0					
Alcaligenaceae	*Achromobacter*	0.0	1.3	0.0					
Pseudomonadaceae	*Pseudomonas*	0.0	1.0	−0.1					
Rhodobacteraceae	*Rubellimicrobium*	0.0	1.0	0.0					

*Activity values of each genus in upper medium, lower medium and lower pooled fractions is showed in Umed, Lmed and Low columns, respectively*.

* *p < 0.01*;

** *p < 0.05*;

*+ p < 0.1*.

### PICRUSt predicted KEGG ortholog enrichment in dense isotopically labeled fractions

After running PICRUSt, the predicted metagenome of each dense pooled fraction (medium and lower) from the isotopically labeled gradient (^13^C or ^15^N) was compared to that in the control gradient (^12^C or ^14^N). Heatmaps were constructed showing the enrichment or underrepresentation of KEGG Orthologs dense pooled fractions of the isotopically labeled gradients, as an indication of its presence or absence among isotopically labeled bacterial families.

We considered 39 KEGG Orthologs involved in lignocellulose degradation for insects fed with a diet supplemented with cellulose (Supplementary Figure 3). Only the most significantly active families detected by Illumina-SIP were considered in the metagenomic prediction (Lachnospiraceae and Enterococcaceae in larvae, Enterobacteriaceae in adults). KEGG Orthologs involved in cellulose and hemicellulose degradation were the most enriched in labeled lower pooled fraction in both larvae and adults, suggesting that the cellulose-labeled families might be able to process hemicellulose as well. Some KEGG Orthologs potentially involved in lignin digestion were present only in the labeled bacterial family from adults (Enterobacteriaceae).

For insects fed with a diet supplemented with urea, we considered 49 KEGG Orthologs involved in nitrogenous waste degradation (uric acid and urea) and in the production of amino acids from ammonia (NH_3_), the end product of uric acid and urea degradation, along with CO_2_ (Supplementary Figure 4). Only the most significantly active bacterial families detected by Illumina-SIP were considered in the metagenomic prediction (Burkholderiaceae in larvae, Porphyromonadaceae in adults). The pathways leading to the synthesis of amino acids from free ammonia appear to be enriched in urea-labeled bacteria from adults and larvae, supporting their ability to incorporate waste nitrogen back into amino acids. The uricolytic and ureolytic pathways, however, appear to be only barely enriched in labeled bacterial families from larvae. This brings up the possibility of a cross-feeding between ammonia and amino acid producers.

### Determining the level of isotopic enrichment of insect tissue

**Figure 6:**
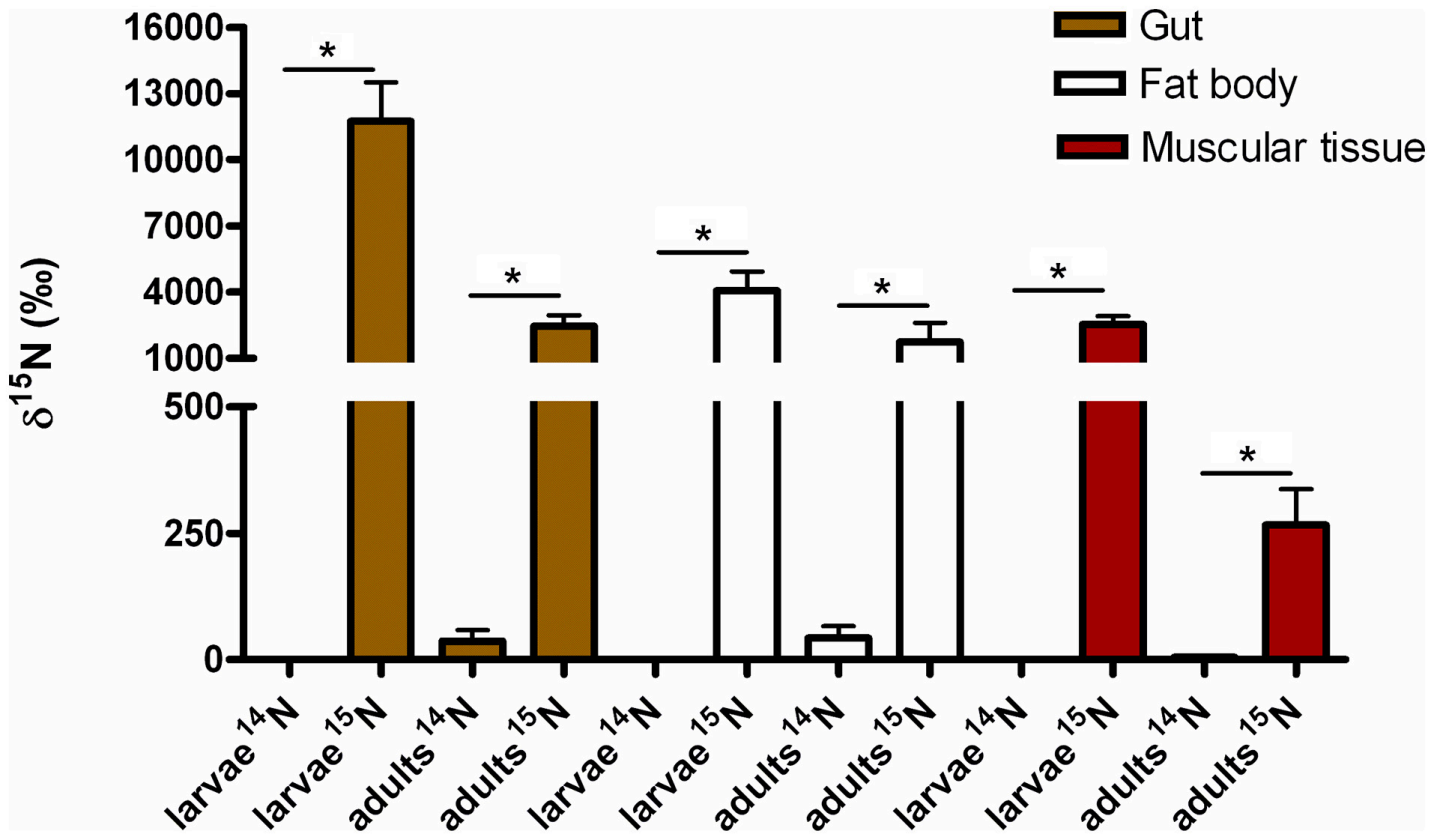
^15^N incorporated into body tissues of *M. hippocastani* reared with diet supplemented with ^15^N urea compared with insects reared with diet supplemented with ^14^N urea. Results shown are mean values for three replicates per treatment. Error bars represent the standard error of the mean, and asterisks represent statistically significant values with *p* = 0.05.

Isotope Ratio Mass Spectrometry (IRMS) measurements of selected insect tissues (gut, fat bodies and muscular tissue) showed the statistically significant incorporation of ^15^N into the insect bodies of both larvae and adults (Figure 6), suggesting host ability to reuse waste nitrogen in both life stages.

## Discussion

### Symbiotic community involved in the processing of cellulose in larvae and adults

Symbiotic bacterial families involved in the processing of cellulose were detected by combining data from SIP and Illumina MiSeq. The less evident DNA density shift in cellulose gradients compared to urea is probably due to the lower speed and longer centrifugation time used (Cadisch et al., 2005), coupled with the higher resolution achieved by splitting urea gradients in 24 fractions instead of 12. As hypothesized in the introduction, Illumina sequencing results showed that the taxonomic affiliation of the active community depended on the stage of insect development, and it involved not only dominant bacterial groups but also minor ones. Influence of soil environmental bacteria on the observed shift is negligible, as gut community composition of *M. hippocastani* larvae shows no overlap with that of the surrounding soil (Arias-Cordero et al., 2012). By feeding the insects during 5 days, we aimed to capture as much as possible of the active community involved in the trophic network of cellulose degradation, not only the primary consumers. When interpreting the results, the limitations of SIP must be taken into account: non-detectable labeling of a certain bacterial family may indicate that they are not able to use cellulose or any of its degradation products, but also it might be a consequence of insufficient label incorporation due to (a) slow growth or (b) reliance on other carbon sources besides cellulose. In larvae, the families Lachnospiraceae, Enterococcaceae, and Desulfovibrionaceae showed the highest activities in terms of cellulose processing (Figure 2). These are anaerobic, fermentative families, although some Desulfovibrionaceae and Enterococcaceae members are able to cope with oxygen (Cypionka, 2000; Felis et al., 2015). The Lachnospiraceae family display cellulolytic capabilities (Chatterjee et al., 2015), and its abundance in the gut of the scarabaeid *Holotrichia parallela* is enriched by woody diets (Huang et al., 2013). Moreover, genomic analyses unveiled presence of butyrate production genes in this family (Vital et al., 2014) and evidence points to them as potential acetogens (Gagen et al., 2015). Thus, the Lachnospiraceae family probably contributes to the fatty acid pool of the gut. Cellulases are produced by Enteroccoccaceae members as well (Shil et al., 2014), and it has been demonstrated that presence of *Enterococcus faecalis* in the digestive tract increases the amount of food consumed by a beetle host, presumably due to its cellulolytic activity (Schmid et al., 2014). Short-chain fatty acids resulting of fermentative processes, such as acetate, propionate or butyrate, may be absorbed through the gut epithelium, and used by the insect as energy source via citrate cycle or carbon source for the synthesis of fatty acids (Bayon, 1980; Bayon and Mathelin, 1980; Odelson and Breznak, 1983; Terra and Ferreira, 2009). Desulfovibrionaceae might have incorporated some labeling due to cross-feeding interaction with primary cellulose consumers, as reported for termite guts, where members of this family oxidize a variety of fermentation products to acetate (Dröge et al., 2005). Desulfovibrionaceae might also enhance cellulose degradation by balancing the redox potential of the gut content and contributing to the maintenance of anoxic conditions (Bharati et al., 1982; Dröge et al., 2005; Arias-Cordero et al., 2012). Collectively, these observations suggest that the families Lachnospiraceae, Enterococcaceae, and Desulfovibrionaceae might work in synergy in the larval gut, Lachnospiraceae and Enterococcaceae breaking down root organic substrates and making them suitable for the host's digestion, absorption and metabolism, and Desulfovibrionaceae maintaining optimal conditions for this process. Host-produced cellulases released in the midgut may also participate in cellulose degradation (Watanabe and Tokuda, 2010; Calderon-Cortes et al., 2012), although the short residence time of the food in the midgut (4–8 h in *M. melolontha*) compared to the hindgut (up to 4 days) (Wildbolz, 1954) suggest that the bulk of digestion of recalcitrant compounds is carried out in the hindgut. Although the families Lachnospiraceae and Desulfovibrionaceae are abundant in the larval gut (Egert et al., 2005; Arias-Cordero et al., 2012), Enterococcaceae seems to be minor (Alonso-Pernas et al., 2017). This reflects the role of secondary bacterial phylotypes, which are easily overlooked, in major gut processes such as cellulose digestion. At the genus level, *Trabulsiella* sp. (Enterobacteriaceae family) showed the highest activity of the identified genera (Table 1). The type species of this genus, *Trabulsiella guamensis*, shows ability to ferment a wide variety of carbohydrates, including glucose and cellobiose (Janda, 2006), and genomic analyses of five strains of *Trabulsiella* sp. isolated from the wood-feeding termite *Heterotermes* sp. showed the presence of an array of cellulolytic enzymes, suggesting their involvement in cellulose degradation within the digestive tract (Olvera-García et al., 2015).

In adults, a much less diverse community made up of exclusively the Enterobacteriaceae family (Figure 3) was significantly active in the processing of cellulose. At the family level, Enterobacteriaceae was not active in larvae. This family, which is commonly associated with herbivore insects, possesses broad polysaccharide-degrading abilities, including cellulose, pectins, xylan and starch (Anand et al., 2010; Adams et al., 2011; Engel et al., 2012), and yield succinate, acetate, H_2_ and CO_2_, among other fermentation products (Wüst et al., 2011). As stated before, acetate can be taken up through the gut epithelium and used by the host (Bayon and Mathelin, 1980; Terra and Ferreira, 2009). The involvement of Enterobacteriaceae members in digestive processes during adult stage is not surprising given the increase in abundance of this bacterial family in the adult gut, where it becomes one of the dominant taxa (Arias-Cordero et al., 2012; Alonso-Pernas et al., 2017). The PICRUSt predicted metagenome shows that the Enterobacteriaceae family might be behind the digestion of a wide array of recalcitrant compounds in the adult gut (Supplementary Figure 3). This simplification of the cellulolytic bacterial network in adults compared to larvae may be a consequence of the increased palatability of a foliage-based diet (32 mg lignin/g dry weight, in oak) compared to a root-based diet (128 mg lignin/g dry weight, in oak) (Arias-Cordero et al., 2012). Among the identified genera, *Enterococcus* sp. (Enterococcaceae family) showed the highest activity, yet it was not statistically significant, suggesting that the most active genus in cellulose degradation within the adult gut may remain unidentified (Table 1). As discussed previously for larvae, cellulolytic *Enterococcus* spp. are important for the nourishment of the host (Schmid et al., 2014; Shil et al., 2014). *Enterococcus mundtii*, which is phylogenetically closely related to our *Enterococcus* sp. (Supplementary Figure 2), has been identified as the most active bacterium in the gut of the leaf-feeding larvae of *Spodoptera littoralis* (Shao et al., 2014).

### Symbiotic community involved in the processing of urea in larvae and adults

Urea is a secondary insect nitrogenous waste compound and also a degradation product of the main compound, uric acid (Bursell, 1967; Ayayee et al., 2014). Thus, by using urea as trophic link, we aimed to target not only urea but also some uric acid degraders within the gut symbiotic community. Additionally, because of long exposition to labeled urea (7 days), we expected to extend the isotopic labeling to secondary substrate consumers, namely amino acid producers, for a better understanding of the trophic network behind the recycling of nitrogen. Labeling was successful in both larvae and adults, indicating the ability of gut bacteria to break down urea and incorporate the heavy isotope into their DNA. Also, IRMS analyses detected the significant incorporation of ^15^N in insect tissues (Figure 6). Taken together, these results strongly indicate the existence of a symbiont-driven nitrogen recycling mechanism in the gut of *M. hippocastani*, as it happens in planthoppers (Sasaki et al., 1996), termites (Potrikus and Breznak, 1981; Thong-On et al., 2012), and cockroaches (Sabree et al., 2009; Patiño-Navarrete et al., 2014). Isotopic labeling was more prominent and widespread among bacterial taxa in larvae than in adults, which is in line with the lower content of organic nitrogen content in the larval diet (roots) compared to the adult diet (leaves) (Dickson, 1989). In larvae, the Burkholderiaceae family showed the highest activity. The Ruminococcaceae and Christensenellaceae families and the Bacillales order tended to be significantly active at much lower levels (Figure 4). Members of the Burkholderiaceae family are commonly found in association with insects feeding on diets deficient in amino acids, such as wood (Geib et al., 2009) and phloem (Michalik et al., 2016). *Tetraponema* ants harbor Burkholderiales representatives in a pouch-like specialized compartment surrounded by a network of Malphigian tubules, suggesting the involvement of the bacteria in the processing of insect nitrogenous waste (Borm et al., 2008). The role in nitrogen recycling of the other labeled taxa has not yet been studied, to our knowledge. Nevertheless, their low activity may indicate a cross-feeding interaction in which they take up isotopically labeled ammonia excreted by urea-degrading bacteria and use it as nitrogen source for their own growth (Bryant and Robinson, 1961). At the genus level, *Burkholderia* sp. (99% of the Burkholderiaceae family sequences) showed a highly significant activity (*p* < 0.01), pointing it as the most relevant genus within the Burkholderiaceae family (Table 2). This genus has been repeatedly found in the digestive tract of insects thriving on nitrogen-deficient diets (Borm et al., 2008; Grünwald et al., 2009; Kikuchi et al., 2010; Reid et al., 2011; Michalik et al., 2016). Some *Burkholderia* strains exhibit nitrogen-fixing ability (Estrada-de los Santos et al., 2001), and the genome of a *Burkholderia*-belonging symbiont encodes complete metabolic pathways for essential amino acids (Shibata et al., 2013). Collectively, these findings suggest *Burkholderia* sp. as a significant bacterium for the recycling of nitrogen in larval *M. hippocastani*.

A change in the active families between larvae and adults indicates that the taxonomic shift is not exclusive to bacteria involved in cellulose digestion, but also happens to bacteria involved in urea processing. The Porphyromonadaceae family showed the highest activity in adults, followed by Bacteroidaceae. Tendency to be significantly active is observed in the family Ruminococcaceae (Figure 5). An increase in abundance of the Porphyromonadaceae family and the order Bacteroidales, to which Porphyromonadaceae and Bacteroidaceae belong, has been observed in the hindgut wall of *M. hippocastani* adults compared to L2 larvae. The order Bacteroidales appeared as the most abundant taxon in adult hindgut wall, suggesting a highly significant role (Alonso-Pernas et al., 2017). The Porphyromonadaceae family has been linked to nitrogen fixation in the wood-eating beetle *Odontotaenius disjunctus* (Ceja-Navarro et al., 2014). Another study on the cockroach *Blattella germanica* relates low protein content in the diet with an increase in the abundance of this family in the gut (Perez-Cobas et al., 2015). Moreover, a genomic study of a Bacteroidales bacterium, endosymbiont of termite gut protists, unveiled its potential for synthesizing amino acids from ammonia (Hongoh et al., 2008). Taken together, these results suggest the Porphyromonadaceae family as key bacteria under conditions of nitrogen scarcity, with potential to synthesize amino acids. The genus *Parabacteroides* (99% of the Porphyromonadaceae family sequences) showed the highest activity of the identified genera (Table 2). To our knowledge, no report has hitherto addressed the role of this particular genus within the gut, but our data suggest that *Parabacteroides* is the relevant bacterial genus in nitrogenous waste recycling in *M. hippocastani* adults. In second place, the family Bacteroidaceae also showed statistically significant activity. Members of this family might have the genetic potential for the synthesis of amino acids using ammonia from insect waste (Hongoh et al., 2008). In termites belonging to the genus *Reticulitermes, Bacteroides* spp. (Bacteroidaceae family) are found among the bacteria responsible for producing ammonia from insect nitrogenous waste (Potrikus and Breznak, 1981). In the present study all the Bacteroidaceae sequences belong to *Bacteroides* sp. Based on these findings, we point the genus *Parabacteroides* as an important waste nitrogen utilizer within the adult *M. hippocastani* gut, possibily with the aid of *Bacteroides* sp. The putative ability of the family Ruminococcaceae to recycle a host's nitrogenous waste remains, to date, unknown. Their low activity makes plausible that they take some of the free ammonia released by primary urea degraders for their own benefit (Bryant and Robinson, 1961).

Surprisingly, none of the bacterial taxa displaying highest activity values in this study have been identified so far as primary degraders of uric acid or urea. In termites, the families Clostridiaceae, Enterobacteriaceae, Streptococcaceae, and Bacteroidaceae are responsible for uric acid degradation (Potrikus and Breznak, 1981; Thong-On et al., 2012). The order Clostridiales and the family Enterobacteriaceae showed non-significant activities in larvae and adults, respectively (Figures 4, 5). The family Bacteroidaceae achieved significant activity in adults (Figure 5). In line with these results, the PICRUSt outcome shows that KEGG Orthologs involved in uric acid and urea degradation are barely enriched in the medium and lower pooled fractions of the ^15^N gradient in both larvae and adults compared to the ^14^N gradient (Supplementary Figure 4). However, the glutamine synthetase pathway, responsible for the incorporation of free ammonia into glutamate to form glutamine, shows enrichment in both life stages. This might suggest a shared pathway in which urea and uric acid degraders would excrete most of the produced ammonia without incorporating much of the labeled nitrogen, and the bacterial genera discussed above (*Burkholderia* in larvae; *Parabacteroides* and possibly *Bacteroides* in adults) would uptake it for amino- and nucleic acid synthesis. Similar cross-feeding has been reported in a study on the cockroach endosymbiont *Blattabacterium cuenoti*, which degrades urea into ammonia but lacks the ability to use this ammonia through the glutamine synthetase pathway, which is carried out by the host (Patiño-Navarrete et al., 2014). It must be taken into account, however, that such *in silico* predictions are limited by the fact that PICRUSt relies on reference genomes, and does not consider processes such as horizontal gene transfer that may reshape the gene pool of symbiotic bacteria (Hansen and Moran, 2014).

## Concluding remarks

The microbial community inhabiting the digestive tract of *M. hippocastani* is very complex and consequently problematic to study. We showed how SIP combined with Illumina MiSeq (Illumina-SIP) can be used to underscore the bacterial symbionts involved in relevant processes for the host, thus narrowing down the number of bacterial taxa in which future research should focus on. Our experiments were limited to cellulose processing and the recycling of nitrogen, but it is possible to address other functions of the gut microbiome by using different labeled substrates, for instance, the active bacteria involved in host detoxification processes could be assessed with labeled plant defense compounds. Besides highlighting key microbial symbionts committed to the treatment of cellulose and urea, our data unveiled a shift in their taxonomic affiliation depending on host developmental stage, regardless of the observed community stability throughout the entire insect life cycle. Moreover, low-abundance bacterial phylotypes may be of crucial importance for the gut ecosystem, and PICRUSt predictions suggested possible additional roles for the labeled bacteria, such as production of amino acids and digestion of hemicellulose.

This study set up a starting point for research on the function and dymanics of the gut microbial community of *M. hippocastani*, at the same time it opens interesting questions, such as the elucidation of the molecular mechanisms underlying the bacterial taxonomic shift between host larval and adult stages, the confirmation of the *in silico* PICRUSt results with metagenomic data and to deepen into the individual roles of the active bacterial phylotypes.

## Ethics statement

Work with insects does not require approval by an ethics committee.

## Author contributions

PA, EA, AN, and YS designed the experiments. PA and SB performed the experiments. PA, AN, and LH analyzed the data. PA and AN wrote the manuscript. WB conceived and supervised the project.

### Conflict of interest statement

The authors declare that the research was conducted in the absence of any commercial or financial relationships that could be construed as a potential conflict of interest.

## References

[B1] Adams A. S. Jordan M. S. Adams S. M. Suen G. Goodwin L. A. Davenport K. W.. (2011). Cellulose-degrading bacteria associated with the invasive woodwasp *Sirex noctilio*. ISME J. 5, 1323–1331. 10.1038/ismej.2011.14 21368904 PMC3146269

[B2] Alonso-Pernas P. Arias-Cordero E. Novoselov A. Ebert C. Rybak J. Kaltenpoth M.. (2017). Bacterial community and PHB-accumulating bacteria associated with the wall and specialized niches of the hindgut of the forest cockchafer (*Melolontha hippocastani*). Front. Microbiol. 8:291. 10.3389/fmicb.2017.00291 28293223 PMC5329036

[B3] Anand A. P. Vennison S. J. Sankar S. G. Prabhu D. I. G. Vasan P. T. Raghuraman T.. (2010). Isolation and characterization of bacteria from the gut of *Bombyx mori* that degrade cellulose, xylan, pectin and starch and their impact on digestion. J. Insect Sci. 10, 107. 10.1673/031.010.10701 20874394 PMC3016902

[B4] Aoyagi T. Hanada S. Itoh H. Sato Y. Ogata A. Friedrich M. W.. (2015). Ultra-high-sensitivity stable-isotope probing of rRNA by high-throughput sequencing of isopycnic centrifugation gradients. Environ. Microbiol. Rep. 7, 282–287. 10.1111/1758-2229.12243 25403652

[B5] Arias-Cordero E. Ping L. Reichwald K. Delb H. Platzer M. Boland W. (2012). Comparative evaluation of the gut microbiota associated with the below- and above-ground life stages (larvae and beetles) of the forest cockchafer, *Melolontha hippocastani*. PLoS ONE 7:1557. 10.1371/journal.pone.0051557 23251574 PMC3519724

[B6] Ayayee P. Rosa C. Ferry J. G. Felton G. Saunders M. Hoover K. (2014). Gut microbes contribute to nitrogen provisioning in a wood-feeding cerambycid. Environ. Entomol. 43, 903–912. 10.1603/EN14045 24937261

[B7] Baumberger J. P. (1919). A nutritional study of insects, with special reference to microorganisms and their substrata. J. Exp. Zool. 28, 1–81. 10.1002/jez.1400280102

[B8] Bayon C. (1980). Volatile fatty acids and methane production in relation to anaerobic carbohydrate fermentation in Oryctes nasicornis larvae (Coleoptera:Scarabaeidae). J. Insect Physiol. 26, 819–828. 10.1016/0022-1910(80)90098-0

[B9] Bayon C. Mathelin J. (1980). Carbohydrate fermentation and by-product absortion studied with labelled cellulose in *Oryctes nasicornis* larvae (Coleoptera:Scarabaeidae). J. Insect Physiol. 26, 833–840. 10.1016/0022-1910(80)90100-6

[B10] Bell T. H. Yergeau E. Martineau C. Juck D. Whyte L. G. Greer C. W. (2011). Identification of nitrogen-incorporating bacteria in petroleum-contaminated arctic soils by using [15N]DNA-based stable isotope probing and pyrosequencing. Appl. Environ. Microbiol. 77, 4163–4171. 10.1128/AEM.00172-11 21498745 PMC3131650

[B11] Berry D. Stecher B. Schintlmeister A. Reichert J. Brugiroux S. Wild B.. (2013). Host-compound foraging by intestinal microbiota revealed by single-cell stable isotope probing. Proc. Natl. Acad. Sci. U.S.A. 110, 4720–4725. 10.1073/pnas.1219247110 23487774 PMC3607026

[B12] Bharati P. L. Baulaigue R. Matheron R. (1982). Degradation of cellulose by mixed cultures of fermentative bacteria and anaerobic sulfur bacteria. Zentralblatt Fur Bakteriol. Mikrobiol. Und Hyg. 3, 466–474. 10.1016/S0721-9571(82)80003-3

[B13] Böhlke J. Coplen T. (1995). Interlaboratory comparison of reference materials for nitrogen-isotope-ratio measurements. IAEA-TECDOC 825, 51–62.

[B14] Borm S. Van Buschinger A. Boomsma J. J. (2008). *Tetraponera* ants have gut symbionts related to nitrogen-fixing root-nodule bacteria. Hung. Q. 49, 2023–2027. 10.1098/rspb.2002.2101 PMC1691126 12396501

[B15] Brand W. A. Coplen T. B. (2012). Stable isotope deltas: tiny, yet robust signatures in nature. Isotopes Environ. Health Stud. 48, 393–409. 10.1080/10256016.2012.666977 22462621

[B16] Bridges J. R. (1981). Nitrogen-fixing bacteria associated with bark beetles. Microb. Ecol. 7, 131–137. 10.1007/BF02032495 24227423

[B17] Bryant M. P. Robinson I. M. (1961). Studies on the nitrogen requirements of some ruminal cellulolytic bacteria. Appl. Microbiol. 9, 96–103. 16349603 10.1128/am.9.2.96-103.1961 PMC1057680

[B18] Bursell E. (1967). The excretion of nitrogen in insects. Adv. In Insect Phys. 4, 33–67. 10.1016/S0065-2806(08)60207-6

[B19] Cadisch G. Espana M. Causey R. Richter M. Shaw E. Morgan J. A. W.. (2005). Technical considerations for the use of 15N-DNA stable-isotope probing for functional microbial activity in soils. Rapid Commun. Mass Spectrom. 19, 1424–1428. 10.1002/rcm.1908 15880617

[B20] Calderon-Cortes N. Quesada M. Watanabe H. Cano-Camacho H. Oyama K. (2012). Endogenous plant cell wall digestion : a key mechanism in insect evolution. Ann. Rev. Ecol. Evol. Syst. 43, 45–71. 10.1146/annurev-ecolsys-110411-160312

[B21] Caporaso J. G. Bittinger K. Bushman F. D. Desantis T. Z. Andersen G. L. Knight R. (2010a). PyNAST: A flexible tool for aligning sequences to a template alignment. Bioinformatics 26, 266–267. 10.1093/bioinformatics/btp636 19914921 PMC2804299

[B22] Caporaso J. G. Kuczynski J. Stombaugh J. (2010b). QIIME allows analysis of high-throughput community sequencing data. Nat. Methods 7, 335–336. 10.1038/nmeth.f.303 20383131 PMC3156573

[B23] Ceja-Navarro J. A. Nguyen N. H. Karaoz U. Gross S. R. Herman D. J. Andersen G. L.. (2014). Compartmentalized microbial composition, oxygen gradients and nitrogen fixation in the gut of *Odontotaenius disjunctus*. ISME J. 8, 6–18. 10.1038/ismej.2013.134 23985746 PMC3869013

[B76] Chatterjee S. Sharma S. Prasad R. K. Datta S. Dubey D. Meghvansi M. K. (2015). Cellulase enzyme based biodegradation of cellulosic materials: an overview. South Asian J. Exp. Biol. 5, 271–282.

[B24] Cleveland L. (1924). The physiological and symbiotic relationships between the intestinal protozoa of termites and their host, with special reference to *Reticulitermes flavipes* kollar. Biol. Bull. 46, 117–227.

[B25] Colman D. R. Toolson E. C. Takacs-Vesbach C. D. (2012). Do diet and taxonomy influence insect gut bacterial communities? Mol. Ecol. 21, 5124–5137. 10.1111/j.1365-294X.2012.05752.x 22978555

[B26] Cypionka H. (2000). Oxygen respiration by *Desulfovibrio* species. Ann. Rev. Microbiol. 54, 827–48. 10.1146/annurev.micro.54.1.827 11018146

[B27] Dickson R. E. (1989). Carbon and nitrogen allocation in trees. Ann. Des. Sci. Fores. 46, 631s–647s. 10.1051/forest:198905ART0142

[B28] Douglas A. E. (2009). The microbial dimension in insect nutritional ecology. Funct. Ecol. 23, 38–47. 10.1111/j.1365-2435.2008.01442.x

[B29] Dröge S. Limper U. Emtiazi F. Schönig I. Pavlus N. Drzyzga O.. (2005). *In vitro* and *in vivo* sulfate reduction in the gut contents of the termite *Mastotermes darwiniensis* and the rose chafer *Pachnoda marginata*. J. Gen. Appl. Microbiol. 51, 57–64. 10.2323/jgam.51.57 15942866

[B30] Dumont M. G. Murrell J. C. (2005). Stable isotope probing — linking microbial identity to function. Nat. Rev. Microbiol. 3, 499–504. 10.1038/nrmicro1162 15886694

[B31] Edgar R. C. (2010). Search and clustering orders of magnitude faster than BLAST. Bioinformatics 26, 2460–2461. 10.1093/bioinformatics/btq461 20709691

[B32] Egert M. Stingl U. Bruun L. D. Brune A. Friedrich M. W. Pommerenke B. (2005). Structure and topology of microbial communities in the major gut compartments of *Melolontha melolontha* larvae (Coleoptera : Scarabaeidae). Appl. Environ. Microbiol. 71, 4556–4566. 10.1128/AEM.71.8.4556-4566.2005 16085849 PMC1183286

[B33] Egert M. Wagner B. Lemke T. Brune A. Friedrich M. W. (2003). Microbial community structure in midgut and hindgut of the humus-feeding larva of *Pachnoda ephippiata* (Coleoptera : Scarabaeidae). Appl. Environ. Microbiol. 69, 6659–6668. 10.1128/AEM.69.11.6659-6668.2003 14602626 PMC262301

[B34] Engel P. Moran N. A. (2013). The gut microbiota of insects – diversity in structure and function. FEMS Microbiol. Rev. 37, 699–735. 10.1111/1574-6976.12025 23692388

[B35] Engel P. Martinson V. G. Moran N. A. (2012). Functional diversity within the simple gut microbiota of the honey bee. PNAS 109, 11002–11007. 10.1073/pnas.1202970109 22711827 PMC3390884

[B36] Estrada-de los Santos P. Bustillos-Cristales R. Caballero-Mellado J. (2001). *Burkholderia*, a genus rich in plant-associated nitrogen fixers with wide environmental and geographic distribution. Appl. Environ. Microbiol. 67, 2790–2798. 10.1128/AEM.67.6.2790-2798.2001 11375196 PMC92940

[B37] Felis G. Salvetti E. Torriani S. (2015). Systematic of lactic acid bacteria: current status, in Biotechnology of Lactic Acid Bacteria: Novel Applications, eds Mozzi F. Raya R. R. Vignolo G. M. (New York, NY: John Wiley & Sons, Ltd.) 25–32.

[B38] Franzini P. Z. N. Ramond J. Scholtz C. H. Sole C. L. Ronca S. Cowan D. A. (2016). The gut microbiomes of two *Pachysoma* MacLeay desert dung beetle species (Coleoptera: Scarabaeidae:Scarabaeinae) feeding on different diets. PLoS ONE 11:118 10.1371/journal.pone.0161118 PMC4988786 27532606

[B39] Gagen E. J. Padmanabha J. Denman S. E. (2015). Hydrogenotrophic culture enrichment reveals rumen Lachnospiraceae and Ruminococcaceae acetogens and hydrogen-responsive Bacteroidetes from pasture-fed cattle. FEMS Microbiol. Lett. 362, 1–8. 10.1093/femsle/fnv104 26109360

[B40] Geib S. M. del Mar Jimenez-Gasco M. Carlson J. E. Tien M. Jabbour R. Hoover K. (2009). Microbial community profiling to investigate transmission of bacteria between life stages of the wood-boring beetle, *Anoplophora glabripennis*. Microb. Ecol. 58, 199–211. 10.1007/s00248-009-9501-4 19277770

[B41] Godwin S. Kang A. Gulino L. Manefield M. Kienzle M. Ouwerkerk D.. (2014). Investigation of the microbial metabolism of carbon dioxide and hydrogen in the kangaroo foregut by stable isotope probing. ISME J. 8, 1855–1865. 10.1038/ismej.2014.25 24621520 PMC4139718

[B42] Grünwald S. Pilhofer M. Höll W. (2009). Microbial associations in gut systems of wood- and bark-inhabiting longhorned beetles (Coleoptera: Cerambycidae). Syst. Appl. Microbiol. 33, 25–34. 10.1016/j.syapm.2009.10.002 19962263

[B43] Guerrero E. Ben M. Salvador R. Ceja-navarro J. (2016). Effect of different lignocellulosic diets on bacterial microbiota and hydrolytic enzyme activities in the gut of the cotton boll weevil (*Anthonomus grandis*). Front. Microbiol. 7:2093. 10.3389/fmicb.2016.02093 28082962 PMC5186755

[B44] Hansen A. K. Moran N. A. (2014). The impact of microbial symbionts on host plant utilization by herbivorous insects. Mol. Ecol. 23, 1473–1496. 10.1111/mec.12421 23952067

[B45] Hongoh Y. Sharma V. K. Prakash T. Noda S. Toh H. Taylor T. D.. (2008). Genome of an endosymbiont coupling N2 fixation to cellulolysis within protist cells in termite gut. Science 322, 1108–1109. 10.1126/science.1165578 19008447

[B46] Huang S. Sheng P. Zhang H. (2012). Isolation and identification of cellulolytic bacteria from the gut of *Holotrichia parallela* larvae (Coleoptera: Scarabaeidae). Int. J. Mol. Sci. 13, 2563–2577. 10.3390/ijms13032563 22489111 PMC3317674

[B47] Huang X. Bakker M. G. Judd T. M.. (2013). Variations in diversity and richness of gut bacterial communities of termites (*Reticulitermes flavipes*) fed with grassy and woody Plant substrates. Microb. Ecol. 65, 531–536. 10.1007/s00248-013-0219-y 23529653

[B48] Jackson T. Klein M. G. (2006). Scarabs as pests: a continuing problem. Coleopt. Bull. 60, 102–119. 10.1649/0010-065X(2006)60[102:SAPACP]2.0.CO;2

[B49] Janda J. M. (2006). New members of the family Enterobacteriaceae, in Prokaryotes, eds Dworkin M. Falkow S. Rosenberg E. Schleifer K. H. Stackebrandt E. (Berlin: Springer), 5–40. 10.1007/0-387-30746-X_1

[B50] Jones R. T. Sanchez L. G. Fierer N. (2013). A cross-taxon analysis of insect-associated bacterial diversity. PLoS ONE 8, 1–10. 10.1371/journal.pone.0061218 23613815 PMC3628706

[B51] Kikuchi Y. Hosokawa T. Fukatsu T. (2010). An ancient but promiscuous host – symbiont association between *Burkholderia* gut symbionts and their heteropteran hosts. ISME J. 5, 446–460. 10.1038/ismej.2010.150 20882057 PMC3105724

[B52] Langille M. Zaneveld J. Caporaso J. G. McDonald D. Knights D. Reyes J.. (2013). Predictive functional profiling of microbial communities using 16S rRNA marker gene sequences. Nat. Biotechnol. 31, 814–821. 10.1038/nbt.2676 23975157 PMC3819121

[B53] Liu S. Wawrik B. Liu Z. (2017). Different bacterial communities involved in peptide decomposition between normoxic and hypoxic coastal waters. Front. Microbiol. 8:353. 10.3389/fmicb.2017.00353 28326069 PMC5339267

[B54] Lu Y. Conrad R. (2005). *In situ* stable isotope probing of methanogenic archaea in the rice rhizosphere. Science 309, 1088–1091. 10.1126/science.1113435 16099988

[B55] Lueders T. Manefield M. Friedrich M. W. (2004). Enhanced sensitivity of DNA- and rRNA-based stable isotope probing by fractionation and quantitative analysis of isopycnic centrifugation gradients. Environ. Microbiol. 6, 73–78. 10.1046/j.1462-2920.2003.00536.x 14686943

[B56] Martin M. (1983). Cellulose digestion in insects. Comp. Biochem. Physiol. 75A, 313–324. 10.1016/0300-9629(83)90088-9

[B57] McDonald D. Price M. N. Goodrich J. Nawrocki E. P. DeSantis T. Z. Probst A.. (2012). An improved Greengenes taxonomy with explicit ranks for ecological and evolutionary analyses of bacteria and archaea. ISME J. 6, 610–618. 10.1038/ismej.2011.139 22134646 PMC3280142

[B58] Michalik K. Szklarzewicz T. Kalandyk-Kołodziejczyk M. Jankowska W. Michalik A. (2016). Bacteria belonging to the genus *Burkholderia* are obligatory symbionts of the eriococcids *Acanthococcus aceris* (Signoret, 1875) and *Gossyparia spuria* (Modeer, 1778) (Insecta, Hemiptera, Coccoidea). Arthropod Struct. Dev. 45, 265–272. 10.1016/j.asd.2016.04.002 27109514

[B59] Morales-jiménez J. Vera-Ponce de Leon A. Garcia-Dominguez A. Martinez-Romero E. Zuniga G. Hernandez Rodriguez C. (2013). Nitrogen-fixing and uricolytic bacteria associated with the gut of *Dendroctonus rhizophagus* and *Dendroctonus valens* (Curculionidae : Scolytinae). Microb. Ecol. 66, 200–210. 10.1007/s00248-013-0206-3 23525792

[B60] Neufeld J. D. Vohra J. Dumont M. G. Lueders T. Manefield M. Friedrich M. W.. (2007). DNA stable-isotope probing. Nat. Protoc. 2, 860–866. 10.1038/nprot.2007.109 17446886

[B61] Odelson D. A. Breznak J. A. (1983). Volatile fatty acid production by the hindgut microbiota of xylophagous termites. Appl. Environ. Microbiol. 45, 1602–1613. 16346296 10.1128/aem.45.5.1602-1613.1983 PMC242507

[B62] Olvera-García M. Fontes-Perez H. Chávez-Martínez A. Barrera O. R. Rodríguez-Almeida F. A. Sanchez-Flores A.. (2015). Draft genome sequences for five strains of *Trabulsiella odontotermitis*, isolated from *Heterotermes* sp. termite gut. Genome Announc. 3, e01289–e01215. 10.1128/genomeA.01289-15 26543120 PMC4645205

[B63] Paes F. Liu X. Mattes T. E.. (2015). Elucidating carbon uptake from vinyl chloride using stable isotope probing and Illumina sequencing. Appl. Microbiol. Biotechnol. 99, 7735–7743. 10.1007/s00253-015-6606-1 25981993

[B64] Patiño-Navarrete R. Piulachs M.-D. Belles X. Moya A. Latorre A. Peretó J. (2014). The cockroach *Blattella germanica* obtains nitrogen from uric acid through a metabolic pathway shared with its bacterial endosymbiont. Biol. Lett. 10, 7–10. 10.1098/rsbl.2014.0407 25079497 PMC4126632

[B65] Perez-Cobas A. E. Maiques E. Angelova A. Carrasco P. Moya A. Latorre A. (2015). Diet shapes the gut microbiota of the omnivorous cockroach *Blattella germanica*. FEMS Microbiol. Ecol. 91, 1–14. 10.1093/femsec/fiv022 25764470

[B66] Potrikus C. J. Breznak J. A. (1981). Gut bacteria recycle uric acid nitrogen in termites: a strategy for nutrient conservation. Proc. Natl. Acad. Sci. U.S.A. 78, 4601–4605. 10.1073/pnas.78.7.4601 16593064 PMC319841

[B67] Reeder J. Knight R. (2010). Rapidly denoising pyrosequencing amplicon reads by exploiting rank-abundance distributions. Nat. Methods 7, 668–669. 10.1038/nmeth0910-668b 20805793 PMC2945879

[B68] Reichardt N. Barclay A. R. Weaver L. T. Morrison D. J. (2011). Use of stable isotopes to measure the metabolic activity of the human intestinal microbiota. Appl. Environ. Microbiol. 77, 8009–8014. 10.1128/AEM.05573-11 21948826 PMC3208973

[B69] Reid N. M. Addison S. L. Macdonald L. J. Lloyd-Jones G. (2011). Biodiversity of active and inactive bacteria in the gut flora of wood-feeding huhu beetle larvae (*Prionoplus reticularis*). Appl. Environ. Microbiol. 77, 7000–7006. 10.1128/AEM.05609-11 21841025 PMC3187079

[B70] Rössler M. (1961). Ernährungsphysiologische Untersuchungen an *Scarabaeiden larven* (*Oryctes nasicornis* L., *Melolontha melolontha* L.). J. Insect Physiol. 6, 62–80. 10.1016/0022-1910(61)90092-0

[B71] Sabree Z. L. Kambhampati S. Moran N. A. (2009). Nitrogen recycling and nutritional provisioning by *Blattabacterium*, the cockroach endosymbiont. Proc. Natl. Acad. Sci. U.S.A. 106, 19521–19526. 10.1073/pnas.0907504106 19880743 PMC2780778

[B72] Saeed A. Sharov V. White J. Li J. Liang W. Bhagabati N.. (2003). TM4: a free, open-source system for microarray data management and analysis. BioTechniques 34, 374–378. 12613259 10.2144/03342mt01

[B73] Sasaki T. Kawamura M. Ishikawa H. (1996). Nitrogen recycling in the brown planthopper, *Nilaparvata lugens*: Involvement of yeast-like endosymbionts in uric acid metabolism. J. Insect Physiol. 42, 125–129. 10.1016/0022-1910(95)00086-0

[B74] Schmid R. B. Lehman R. M. Brözel V. S. Lundgren J. G. (2014). An indigenous gut bacterium, *Enterococcus faecalis* (Lactobacillales : Enterococcaceae), increases seed consumption by *Harpalus pensylvanicus* (Coleoptera : Carabidae). Florida Entomol. 97, 575–584. 10.1653/024.097.0232

[B75] Shao Y. Arias-Cordero E. Guo H. Bartram S. Boland W. (2014). *In vivo* Pyro-SIP assessing active gut microbiota of the cotton leafworm, *Spodoptera littoralis*. PLoS ONE 9:5948. 10.1371/journal.pone.0085948 24475063 PMC3903505

[B77] Shi W. Syrenne R. Sun J. Yuan J. S. (2010). Molecular approaches to study the insect gut symbiotic microbiota at the omics age. Insect Sci. 17, 199–219. 10.1111/j.1744-7917.2010.01340.x

[B78] Shibata T. F. Maeda T. Nikoh N. Yamaguchi K. Oshima K. Hattori M.. (2013). Complete genome sequence of *Burkholderia* sp. strain RPE64, bacterial symbiont of the bean bug *Riptortus pedestris*. Genome Announc. 1, e00441–e00413. 10.1128/genomeA.00441-13 23833137 PMC3703598

[B79] Shil R. K. Mojumder S. Sadida F. F. Uddin M. Sikdar D. (2014). Isolation and identification of cellulolytic bacteria from the gut of three phytophagus insect species. Braz. Arch. Biol. Technol. 57, 927–932. 10.1590/S1516-8913201402620

[B80] Stubner S. (2002). Enumeration of 16S rDNA of *Desulfotomaculum* lineage 1 in rice field soil by real-time PCR with SybrGreen detection. J. Microbiol. Methods 50, 155–164. 10.1016/S0167-7012(02)00024-6 11997166

[B81] Tamura K. Stecher G. Peterson D. Filipski A. Kumar S. (2013). MEGA6: Molecular evolutionary genetics analysis version 6.0. Mol. Biol. Evol. 30, 2725–2729. 10.1093/molbev/mst197 24132122 PMC3840312

[B82] Terra W. Ferreira C. (2009). Digestive system, in Encyclopedia of Insects, eds Resh V. Carde R. (Cambridge, MA: Elsevier), 273–281.

[B83] Thong-On A. Suzuki K. Noda S. Inoue J. Kajiwara S. Ohkuma M. (2012). Isolation and characterization of anaerobic bacteria for symbiotic recycling of uric acid nitrogen in the gut of various termites. Microbes Environ. 27, 186–192. 10.1264/jsme2.ME11325 22791052 PMC4036019

[B84] Vital M. Howe C. Tiedje M. (2014). Revealing the bacterial butyrate synthesis pathways by analyzing (Meta)genomic data. MBio 5, 1–11. 10.1128/mBio.00889-14 24757212 PMC3994512

[B85] Wang Q. Garrity G. M. Tiedje J. M. Cole J. R. (2007). Naïve Bayesian classifier for rapid assignment of rRNA sequences into the new bacterial taxonomy. Appl. Environ. Microbiol. 73, 5261–5267. 10.1128/AEM.00062-07 17586664 PMC1950982

[B86] Watanabe H. Tokuda G. (2010). Cellulolytic systems in insects. Annu. Rev. Entomol. 55, 609–632. 10.1146/annurev-ento-112408-085319 19754245

[B87] Werner R. A. Brand W. A. (2001). Referencing strategies and techniques in stable isotope ratio analysis. Rapid Commun. Mass Spectrom. 15, 501–519. 10.1002/rcm.258 11268135

[B88] White J. R. Nagarajan N. Pop M. (2009). Statistical methods for detecting differentially abundant features in clinical metagenomic samples. PLoS Comput. Biol. 5:352. 10.1371/journal.pcbi.1000352 19360128 PMC2661018

[B89] Wildbolz T. (1954). Beitrag zur anatomie, histologie und physiologie des Darmkanals der Larve von *Melolontha melolontha*. Mitt. schweiz. ent. Ges. Berne 27, 193–240.

[B90] Wüst P. K. Horn M. a Drake H. L. (2011). Clostridiaceae and enterobacteriaceae as active fermenters in earthworm gut content. ISME J. 5, 92–106. 10.1038/ismej.2010.99 20613788 PMC3105676

[B91] Yun J. H. Roh S. W. Whon T. W. Jung M. J. Kim M. S. Park D. S.. (2014). Insect gut bacterial diversity determined by environmental habitat, diet, developmental stage, and phylogeny of host. Appl. Environ. Microbiol. 80, 5254–5264. 10.1128/AEM.01226-14 24928884 PMC4136111

[B92] Zhang H. Jackson T. A. (2008). Autochthonous bacterial flora indicated by PCR-DGGE of 16S rRNA gene fragments from the alimentary tract of *Costelytra zealandica* (Coleoptera: Scarabaeidae). J. Appl. Microbiol. 105, 1277–1285. 10.1111/j.1365-2672.2008.03867.x 18713286

