# Corrigendum: *In Vivo* Isotopic Labeling of Symbiotic Bacteria Involved in Cellulose Degradation and Nitrogen Recycling within the Gut of the Forest Cockchafer (*Melolontha hippocastani*)

**DOI:** 10.3389/fmicb.2018.00488

**Published:** 2018-03-12

**Authors:** Pol Alonso-Pernas, Stefan Bartram, Erika M. Arias-Cordero, Alexey L. Novoselov, Lorena Halty-deLeon, Yongqi Shao, Wilhelm Boland

**Affiliations:** ^1^Department of Bioorganic Chemistry, Max Planck Institute for Chemical Ecology, Jena, Germany; ^2^Department of Evolutionary Neuroethology, Max Planck Institute for Chemical Ecology, Jena, Germany; ^3^Institute of Sericulture and Apiculture, College of Animal Sciences, Zhejiang University, Hangzhou, China

**Keywords:** *Melolontha hippocastani*, nitrogen recycling, cellulose degradation, gut bacteria, symbiotic bacteria, Illumina-SIP, IRMS

In our original research article, there is an error in one of the references cited.

The reference “Sharma, S., Assam, T., Meghvansi, M., Vairale, M., Organis, D., Veer, V., et al. (2015). Cellulase enzyme based biodegradation of cellulosic materials: an overview. *South Asian J. Exp. Biol*. 5, 271–282” should be instead cited as:

“Chatterjee, S., Sharma, S., Prasad, R. K., Datta, S., Dubey, D., Meghvansi M. K., et al. (2015). Cellulase enzyme based biodegradation of cellulosic materials: an overview. *South Asian J. Exp. Biol*. 5, 271–282.”

The authors apologize for the mistake. This error does not change the scientific conclusions of the article in any way.

The original article has been updated.

## Conflict of interest statement

The authors declare that the research was conducted in the absence of any commercial or financial relationships that could be construed as a potential conflict of interest.

