# Supplementary material for: In Vivo Isotopic Labeling of Symbiotic Bacteria Involved in Cellulose Degradation and Nitrogen Recycling within the Gut of the Forest Cockchafer (Melolontha hippocastani)
Source: Front Microbiol. 2017 Oct 12;8:1970. doi: 10.3389/fmicb.2017.01970 (PMC5643479; doi:10.3389/fmicb.2017.01970)
Supplement: Supplementary file 1 [file DataSheet1.DOCX]

Supplementary Material

**In vivo isotopic labeling of symbiotic bacteria involved in cellulose degradation and nitrogen recycling within the gut of the forest cockchafer (*Melolontha hippocastani*)**

**Pol Alonso-Pernas, Stefan Bartram, Erika Arias-Cordero, Alexey Novoselov, Lorena Halty-deLeon, Yongqi Shao, Wilhelm Boland*.**

***Corresponding author:** Wilhelm Boland

Email: boland@ice.mpg.de

## Supplementary Figures.

**
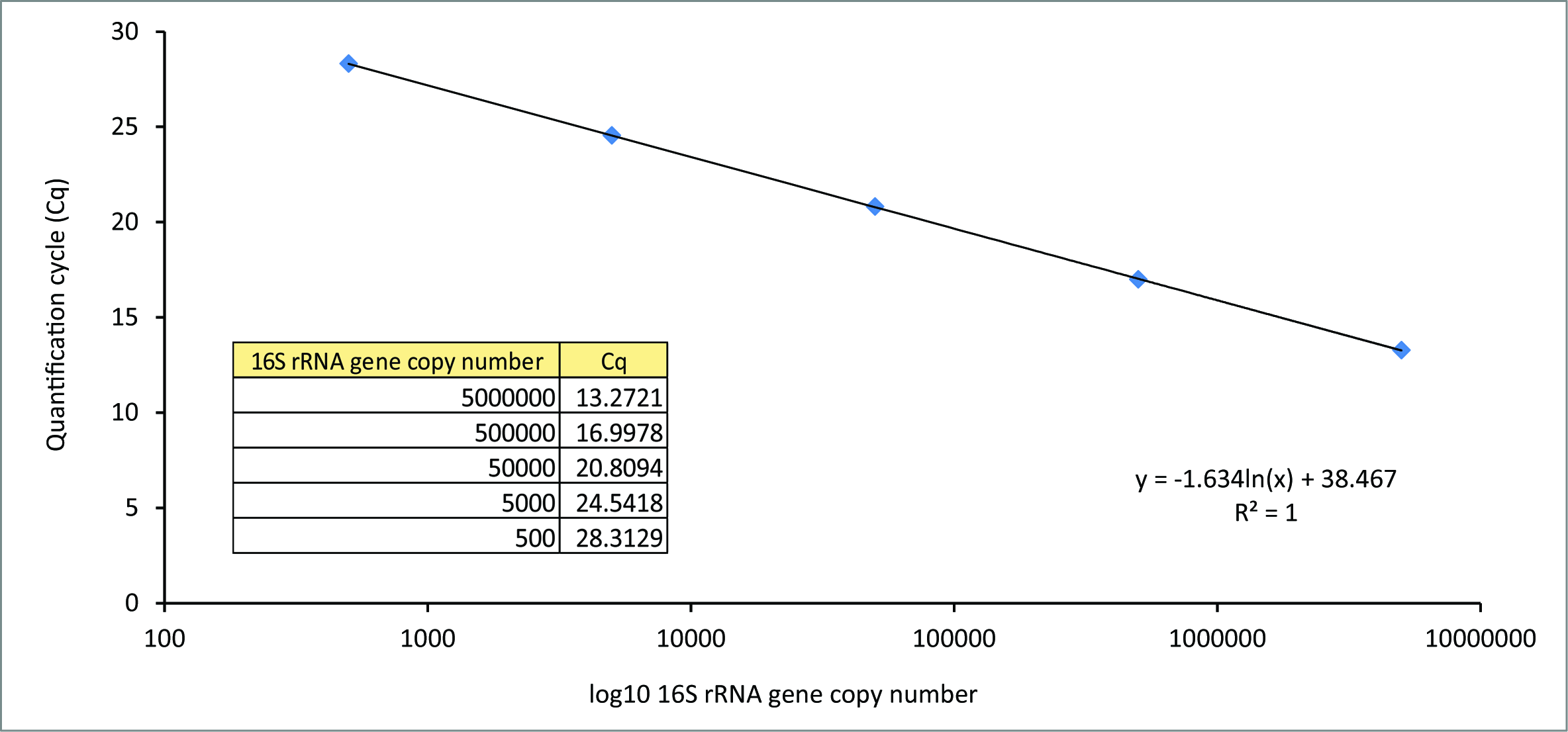
**

**Supplementary Figure 1.** qPCR standard curve constructed using *E.coli* genomic DNA, relating quantitative cycle values to log_10_ transformed 16S rRNA gene copy numbers.


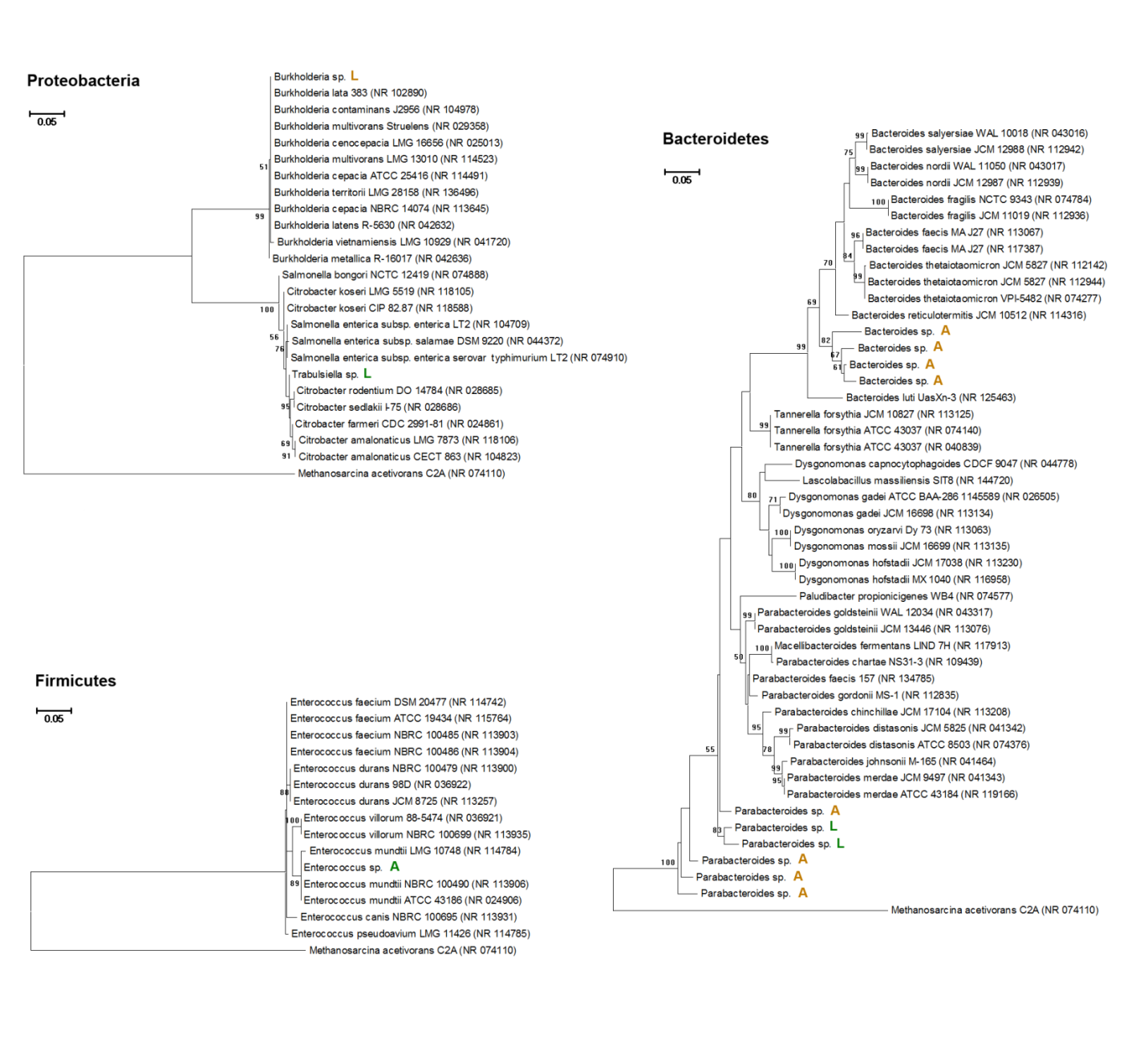


**Supplementary Figure 2.** Maximum likelihood trees relating 16S rDNA sequences of the identified bacterial genera actively involved in the processing of urea and cellulose in *M. hippocastani* gut with their closest BLAST hits in the NCBI database. Active genera detected in the present study are indicated with colored letters A, when coming from adult gut, L, when coming from larval gut. Green indicates that the labeling was done with cellulose, orange indicates that the labeling was done with urea. Reference sequences were downloaded from GenBank (accession numbers are in parentheses). *Methanosarcina acetivorans* (NR 074110) was used as an outgroup. Bootstrap values (in percentages) are based on 1000 replications. Bar represents 5% sequence divergence.

**
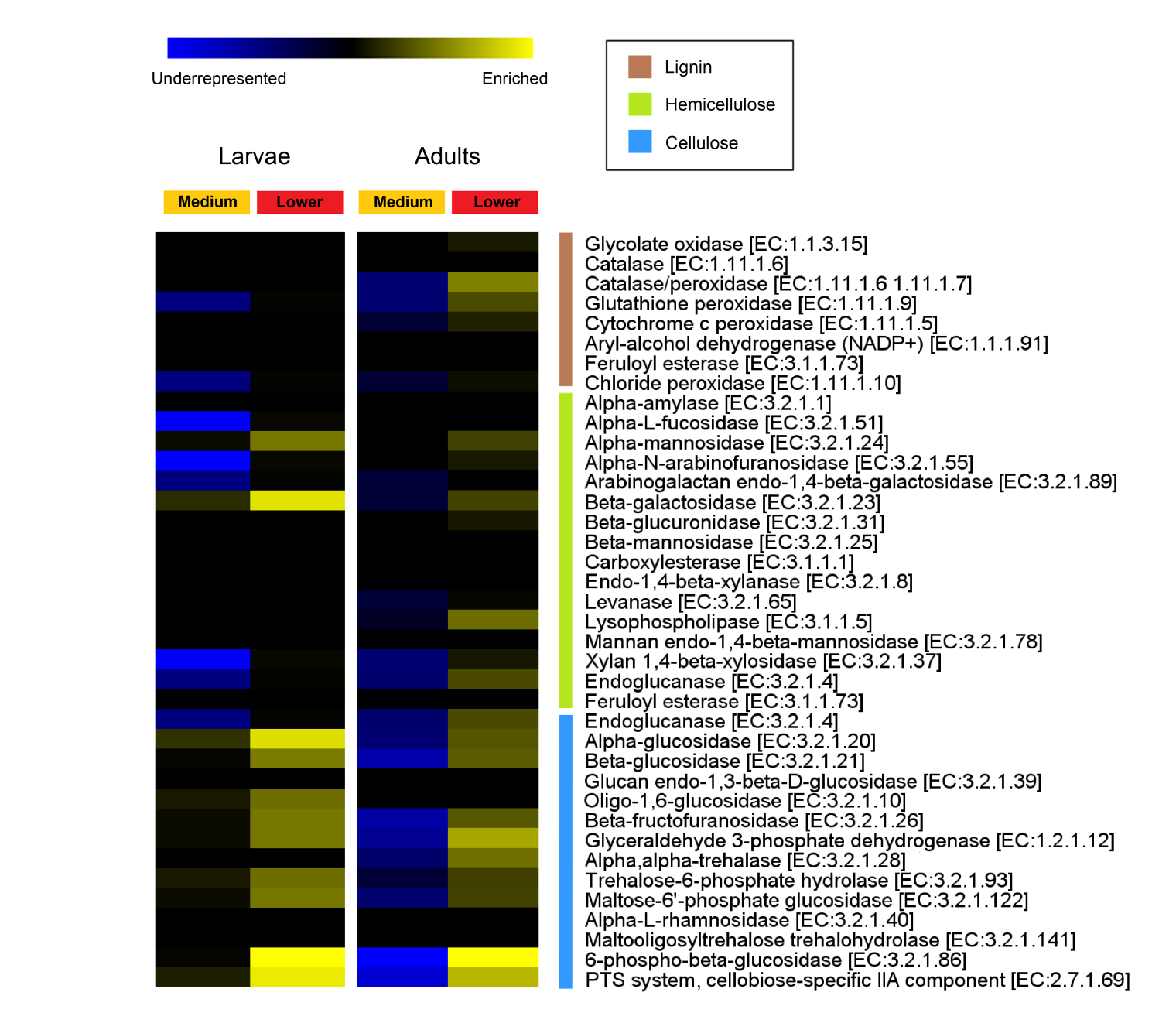
**

**Supplementary Figure 3.** Heatmaps generated after PICRUSt prediction, displaying the enrichment of KEGG Orthologs involved in lignocellulose degradation in the ^13^C medium and lower fractions compared to the same fractions in the ^12^C gradient. The enrichment or underrepresentation of a certain ortholog is an indication of its presence or absence among the isotopically labeled bacterial families (Lachnospiraceae and Enterococcaceae in larvae, Enterobacteriaceae in adults). Blue represents underrepresentation, yellow represents enrichment.

**
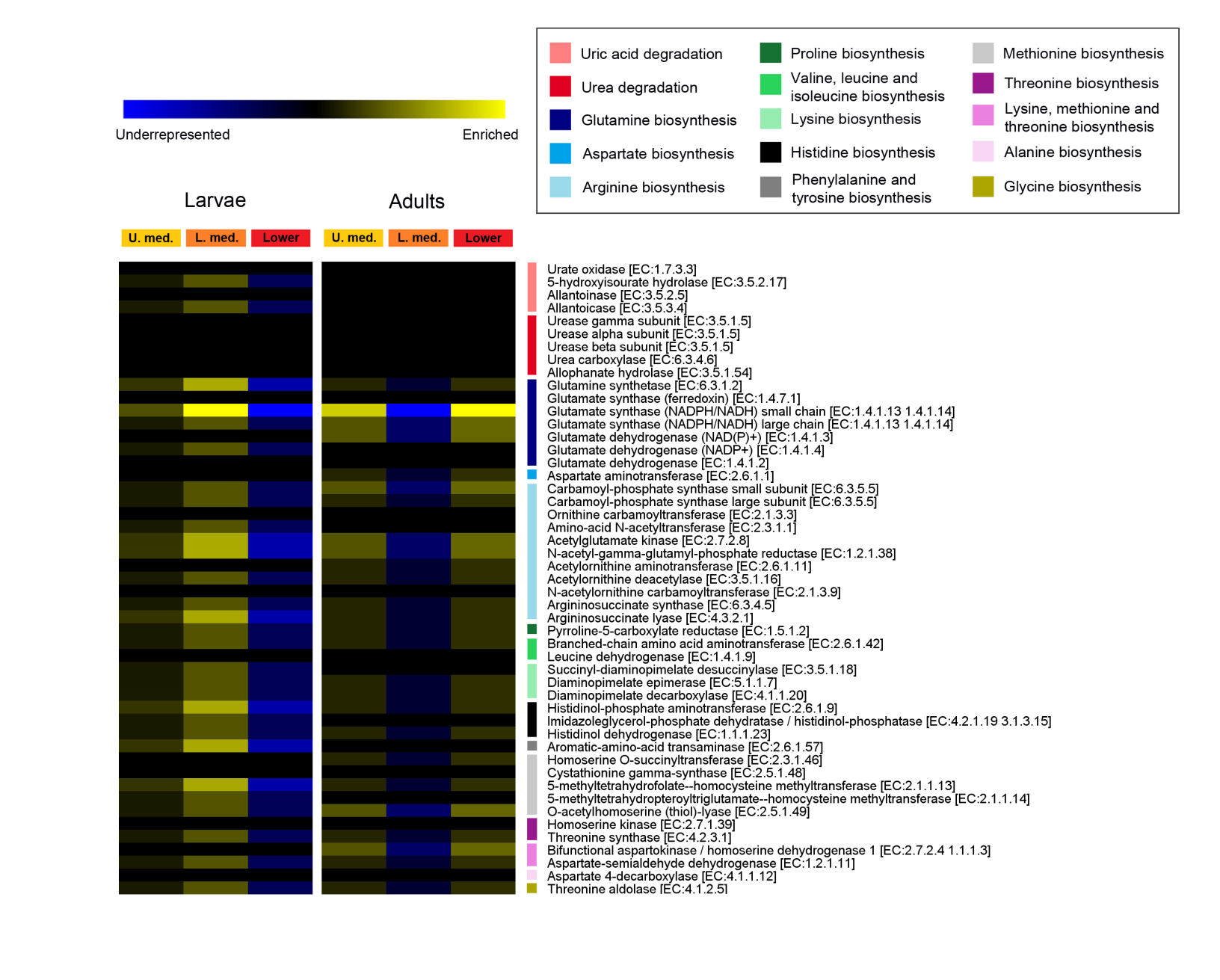
**

**Supplementary Figure 4.** Heatmaps generated after PICRUSt prediction, displaying the enrichment of KEGG Orthologs involved in nitrogenous waste degradation and the synthesis of amino acids in the ^15^N medium and lower fractions compared to the same fractions in the ^14^N gradient. The enrichment or underrepresentation of a certain ortholog is an indication of its presence or absence among the isotopically labeled bacterial families (Burkholderiaceae in larvae, Porphyromonadaceae in adults). Blue represents underrepresentation, yellow represents enrichment.

## Supplementary Tables.

**Supplementary Table 1.** Weighted NSTI values (average branch length (in 16S rRNA substitutions/site) that separates each OTU from a reference bacterial genome, weighted by the abundance of that OTU in the sample) of PICRUSt predicted metagenomes.

| **Gradient fraction** | **Weighted NSTI value** | **Gradient fraction** | **Weighted NSTI value** |
| --- | --- | --- | --- |
| Larvae ^12^C Medium | 0.053388003573 | Larvae ^14^N Upper medium | 0.05254 |
| Larvae ^12^C Lower | 0.0541966375121 | Larvae ^14^N Lower medium | 0.0523413375461 |
| Larvae ^13^C Medium | 0.0605356305385 | Larvae ^14^N Lower | 0.0523634453782 |
| Larvae ^13^C Lower | 0.0600383912423 | Larvae ^15^N Upper medium | 0.052345037037 |
| Adults ^12^C Medium | 0.0501140916073 | Larvae ^15^N Lower medium | 0.0523391317652 |
| Adults ^12^C Lower | 0.075265354104 | Larvae ^15^N Lower | 0.0523584971098 |
| Adults ^13^C Medium | 0.0851468138346 | Adults ^14^N Upper medium | 0.00976 |
| Adults ^13^C Lower | 0.0671953641744 | Adults ^14^N Lower medium | 0.00976 |
|  |  | Adults ^14^N Lower | 0.00976 |
|  |  | Adults ^15^N Upper medium | 0.00976 |
|  |  | Adults ^15^N Lower medium | 0.00976 |
|  |  | Adults ^15^N Lower | 0.00976 |
